# PGE_2_ inhibits TIL expansion by disrupting IL-2 signalling and mitochondrial function

**DOI:** 10.1038/s41586-024-07352-w

**Published:** 2024-04-24

**Authors:** Matteo Morotti, Alizee J. Grimm, Helen Carrasco Hope, Marion Arnaud, Mathieu Desbuisson, Nicolas Rayroux, David Barras, Maria Masid, Baptiste Murgues, Bovannak S. Chap, Marco Ongaro, Ioanna A. Rota, Catherine Ronet, Aspram Minasyan, Johanna Chiffelle, Sebastian B. Lacher, Sara Bobisse, Clément Murgues, Eleonora Ghisoni, Khaoula Ouchen, Ribal Bou Mjahed, Fabrizio Benedetti, Naoill Abdellaoui, Riccardo Turrini, Philippe O. Gannon, Khalil Zaman, Patrice Mathevet, Loic Lelievre, Isaac Crespo, Marcus Conrad, Gregory Verdeil, Lana E. Kandalaft, Julien Dagher, Jesus Corria-Osorio, Marie-Agnes Doucey, Ping-Chih Ho, Alexandre Harari, Nicola Vannini, Jan P. Böttcher, Denarda Dangaj Laniti, George Coukos

**Affiliations:** 1 Ludwig Institute for Cancer Research, Lausanne Branch, University of Lausanne (UNIL), Lausanne, Switzerland; 2 Department of Oncology, Lausanne University Hospital (CHUV) and University of Lausanne, Lausanne, Switzerland; 3 Agora Cancer Research Center, Lausanne, Switzerland; 4 Institute of Molecular Immunology, School of Medicine and Health, Technical University of Munich (TUM), Munich, Germany; 5 Center of Experimental Therapeutics, Department of Oncology, Lausanne University Hospital (CHUV), Lausanne, Switzerland; 6 Department of Gynaecology, Lausanne University Hospital (CHUV), Lausanne, Switzerland; 7 Institute of Metabolism and Cell Death, Molecular Target and Therapeutics Centre, Helmholtz Munich, Neuherberg, Germany; 8 Unit of Translational Oncopathology, Institute of Pathology, Lausanne University Hospital (CHUV), Lausanne, Switzerland

**Keywords:** Tumour immunology, Immunosuppression, Cancer immunotherapy, Tumour immunology

## Abstract

Expansion of antigen-experienced CD8^+^ T cells is critical for the success of tumour-infiltrating lymphocyte (TIL)-adoptive cell therapy (ACT) in patients with cancer^1^. Interleukin-2 (IL-2) acts as a key regulator of CD8^+^ cytotoxic T lymphocyte functions by promoting expansion and cytotoxic capability^2,3^. Therefore, it is essential to comprehend mechanistic barriers to IL-2 sensing in the tumour microenvironment to implement strategies to reinvigorate IL-2 responsiveness and T cell antitumour responses. Here we report that prostaglandin E2 (PGE_2_), a known negative regulator of immune response in the tumour microenvironment^4,5^, is present at high concentrations in tumour tissue from patients and leads to impaired IL-2 sensing in human CD8^+^ TILs via the PGE_2_ receptors EP2 and EP4. Mechanistically, PGE_2_ inhibits IL-2 sensing in TILs by downregulating the IL-2Rγ_c_ chain, resulting in defective assembly of IL-2Rβ–IL2Rγ_c_ membrane dimers. This results in impaired IL-2–mTOR adaptation and PGC1α transcriptional repression, causing oxidative stress and ferroptotic cell death in tumour-reactive TILs. Inhibition of PGE_2_ signalling to EP2 and EP4 during TIL expansion for ACT resulted in increased IL-2 sensing, leading to enhanced proliferation of tumour-reactive TILs and enhanced tumour control once the cells were transferred in vivo. Our study reveals fundamental features that underlie impairment of human TILs mediated by PGE_2_ in the tumour microenvironment. These findings have therapeutic implications for cancer immunotherapy and cell therapy, and enable the development of targeted strategies to enhance IL-2 sensing and amplify the IL-2 response in TILs, thereby promoting the expansion of effector T cells with enhanced therapeutic potential.

## Abstract (web summary)

Prostaglandin E2 from the tumour microenvironment impairs interleukin-2 sensing by tumour-infiltrating lymphocytes, restricting proliferative response and promoting T cell death via metabolic impairment and ferroptosis.

## Main

Adoptive cell therapy (ACT) using autologous TILs has proved to be a powerful and potentially curative therapy in patients with melanoma^6,7^ and is being tested more broadly in solid tumours. Nonetheless, only a fraction of patients with cancer respond to such treatment^8^. Conventional TIL expansion for ACT entails two steps (pre-rapid expansion (pre-REP) followed by rapid expansion (REP)), in which high-dose IL-2 is used to mobilize TILs in tumour fragments from the patient and expand them in culture^6^. During this process, tumour-specific clonotypes may mobilize differently and undergo dilution relative to bystander non-tumour reactive T cells^9^. The successful expansion of relevant cancer-specific TIL clones ultimately determines the potency of TIL-ACT^10^. Although it has long been suspected that local conditions from the native tumour microenvironment (TME) restrain the response to IL-2 in tumour-specific TILs, very little is known about which TME factors drive this restriction.

## PGE_2_ limits TIL mobilization response to IL-2

**Fig. 1:**
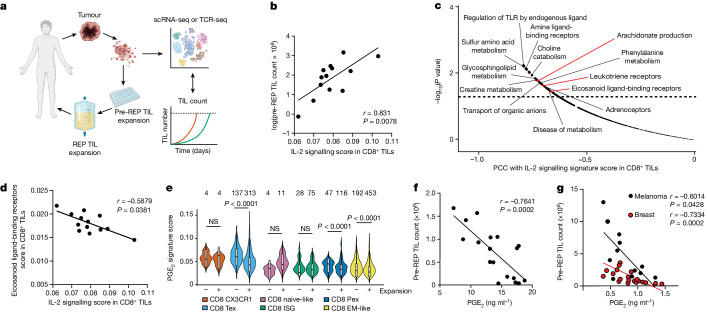
The PGE_2_–EP2/EP4 axis is associated with decreased IL-2-mediated TIL expansion. **a**, Representation of the translational research pipeline of a phase I melanoma TIL-ACT trial. Figure created with BioRender.com. **b**, Correlation between IL-2 signalling score from pseudobulked patient CD8^+^ TILs and total numbers of TILs at pre-REP (on day 11) from patients with melanoma enrolled in the TIL-ACT trial (*n* = 13). **c**, Representation of Reactome pathways that are anti-correlated with IL-2 signalling score from pseudobulked patient CD8^+^ TILs (no correction for multiple testing applied) (*n* = 13). PCC, Pearson’s correlation score. **d**, Correlation between IL-2 signalling score and eicosanoid ligand-binding receptor score from pseudobulked patient CD8^+^ TILs (*n* = 13). **e**, Violin plot of PGE_2_ signature score in tumour-reactive versus non-tumour-reactive CD8^+^ TILs subsets that expanded or did not expand in the cell therapy product. The number of cells is shown at the top of the graph. Box plots display smallest and largest values in the dataset, box hinges represent first and third quartiles with the centre as median and whiskers extend to 1.5× the interquartile range from the first and third quartiles. EM-like, effector memory-like; Pex, precursor exhausted; Tex, terminal exhausted; ISG, IFN-stimulated gene. **f**, Correlation per patient between baseline PGE_2_ levels in the supernatant of expanding TILs from tumour fragments and total numbers of pre-REP TILs in a phase I solid tumour TIL-ACT trial (*n* = 18). **g**, Correlation per patient between PGE_2_ levels in the supernatant of expanding TILs from tumour fragments of breast (*n* = 20) and melanoma (*n* = 12) and total numbers of pre-REP TILs. One-way ANOVA with Tukey’s multiple comparisons test (**e**); two-Sided Spearman’s correlation (**b**,**d**,**f**,**g**); or Pearson’s correlation (**c**). Biological replicates represent individual patients, with exact numbers listed in each panel. NS, not significant (*P* ≥ 0.05).

To investigate the mechanisms affecting IL-2-mediated expansion of tumour-reactive TILs in patients with cancer, we took advantage of coupled single-cell RNA-sequencing (scRNA-seq) and single-cell T cell receptor sequencing (scTCR-seq) analyses conducted in a clinical protocol of TIL-ACT therapy in patients with melanoma^11^ (Fig. 1a). Having paired data from baseline tumours and the products of TIL-ACT, we explored whether the original state of CD8^+^ TILs in tumours in situ affected their propensity to proliferate ex vivo in response to IL-2^11^. We found a positive correlation between a baseline gene signature for IL-2 signalling and the overall TIL expansion (Fig. 1b).

**Extended Data Fig. 1:**
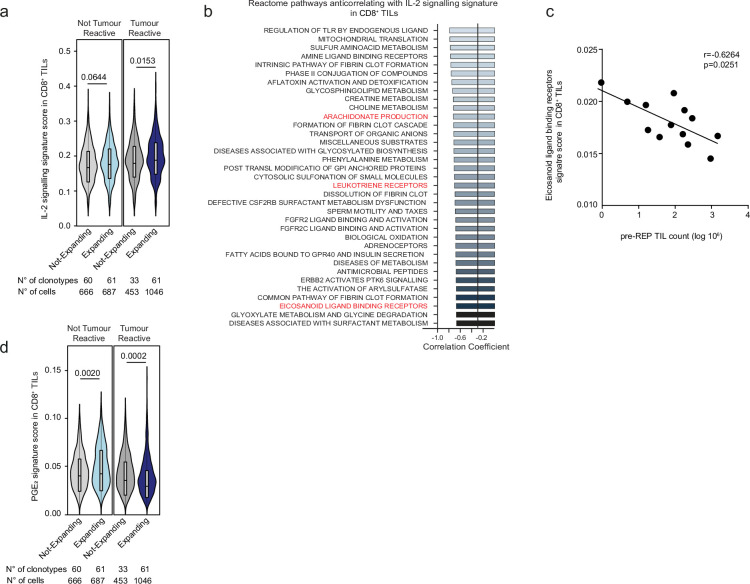
PGE_2_-EP2/EP4 axis is associated with decreased IL-2 mediated TIL expansion. **a**, Violin plot of IL-2 signalling score in tumour reactive / non-tumour reactive CD8^+^ TILs clones that expanded or did not expand in the cell-therapy product. Number of clonotypes and cells are shown. **b**, Top 35 Reactome pathways negatively associated to IL-2 signalling in CD8^+^ TILs from melanoma patients enrolled in the TIL-ACT trial (n = 13). In red are the pathways associated with PGE_2_ signalling. **c**, Correlation per patients between eicosanoid ligand binding receptors score in CD8^+^ TILs and total number of pre-REP TILs (n = 13). **d**, Violin plot of PGE_2_ signature score in tumour reactive/non-tumour reactive CD8^+^ TILs clones that expanded or did not expand in the cell-therapy product. Number of clonotypes and cells are shown. (a,d):Boxplots display smallest and largest values in the dataset. Box hinges: first and third quartile with centre as median. Whiskers: 1.5x interquartile range (IQR) from the first and third quartiles. Statistical comparisons were performed with one-way ANOVA with Tukey’s multiple-comparisons test (a, d) and two-sided Spearman’s correlation (c) or Pearson’s correlation (b). Independent patients were used as biological replicates with exact numbers listed in each panel.

To determine whether this association applied to tumour-specific TILs, we tracked longitudinally—from tumour tissue throughout the ex vivo expansion—215 individual CD8^+^ TIL clonotypes that were previously determined to be tumour-reactive^12^ (Methods). Among the tumour-reactive clonotypes, those that expanded ex vivo exhibited higher IL-2 signalling signature scores in baseline tumours compared with clonotypes that did not expand (Extended Data Fig. 1a). Thus, TILs that are able to sense IL-2 in vivo appear to expand better in IL-2 cultures.

We next sought to the cues in the TME that might impair IL-2 sensing in CD8^+^ TILs in situ. By mining the aforementioned scRNA-seq and TCR-seq data, we uncovered an inverse association between IL-2 signalling and key pathways linked to PGE_2_ production and sensing, including arachidonate production, leukotriene receptors and eicosanoid receptor signalling (Fig. 1c,d and Extended Data Fig. 1b). Similarly, we found a significant association between decreased pre-REP TIL expansion and high eicosanoid ligand-binding receptor score (Extended Data Fig. 1c). These findings suggested that PGE_2_ has a negative effect on IL-2-mediated expansion of human TILs.

**Extended Data Fig. 2:**
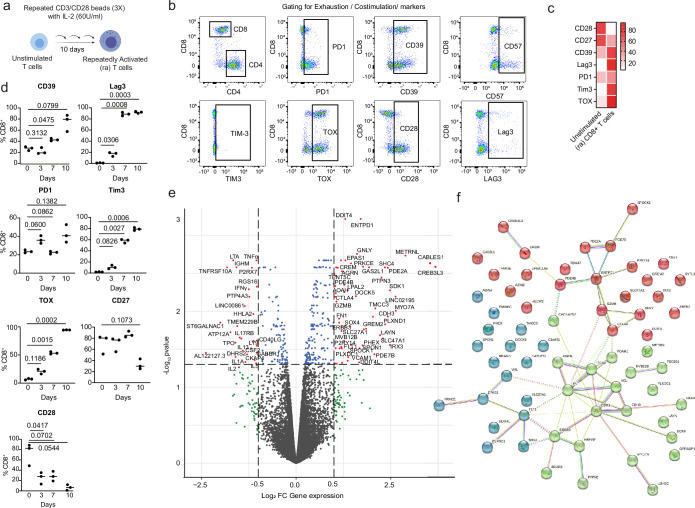
Characterization of repeatedly activated PBLs and PGE_2_-signature. **a**, Schematic representation of the protocol for repeated activation of human peripheral blood lymphocytes using serial CD3/CD28 stimulation every 3 days in low dose IL-2 (Created with BioRender.com). **b**, Representative flow cytometry gates of exhaustion/costimulation markers used to characterize repeatedly activated PBLs. **c**, Heatmap representation of the frequency of inhibitory/co-stimulatory markers and TOX^+^ cells in unstimulated and repeatedly activated CD8^+^ T cells on day 10. **d**, Time-course evaluation of inhibitory markers (CD39, Lag3, PD1, TIM3), exhaustion marker TOX and co-stimulatory molecules (CD27, CD28) expression on CD8^+^ T cells during the repeated activation protocol at day 0-3-7-10. Data are represented as median of 3 biological replicates. Statistical comparisons were performed using one-way ANOVA with Dunnett post-hoc test for multiple comparisons. **e**, Volcano plot of differentially expressed (DE) genes in repeatedly activated CD8^+^ T cells treated with PGE_2_ for 24 h. The Y-axis represents log_10_ p-values, whereas the X-axis represents log_2_ fold change in gene expression. Two-sided paired t test with Benjamini Hochberg correction. **f**, String analysis of the 63 top upregulated genes (p < 0.05, FC > 1) in repeatedly activated CD8^+^ T cells after PGE_2_ treatment (PGE_2_ signature). N = 3 independent biological samples.

To further assess which TIL clonotypes sensed PGE_2_ in situ, we derived a gene signature that revealed recent exposure of human CD8^+^ effector T cells to PGE_2_. To this end, we used an in vitro culture system of repeatedly antigen-stimulated (RA) T cells that phenocopied chronic antigen stimulation and exhaustion (Extended Data Fig. 2a–d), and exposed those cells to PGE_2_ to derive a PGE_2_ signature (Extended Data Fig. 2e,f and Supplementary Table 1). We detected this signature in tumour-reactive CD8^+^ TILs from baseline tumours, in which its expression in tumour-reactive clonotypes correlated with poor ex vivo expansion (Extended Data Fig. 1d). We observed a significant association between increased PGE_2_ exposure signature and lack of ex vivo expansion with IL-2 specifically in CD8^+^ TIL clonotypes that at baseline exhibited precursor-exhausted, terminal-exhausted or effector memory-like states (Fig. 1e)—that is, cellular subsets reported to regroup tumour-specific TIL clones^11^. In agreement, high levels of PGE_2_ produced by tumour fragments in TIL cultures correlated inversely with the outgrowth of TILs from these fragments during pre-REP in an independent cohort of patients undergoing TIL-ACT (Fig. 1f). A similar pattern was observed in two independent cohorts of patients with breast cancer or melanoma (Fig. 1g). Together, our findings from patient-derived tumour tissues suggest a key role for intratumoral PGE_2_ in restraining the expansion capacity of cancer-specific human TILs through regulation of their IL-2 responsiveness.

## PGE_2_ disrupts IL-2 sensing in TILs

**Fig. 2:**
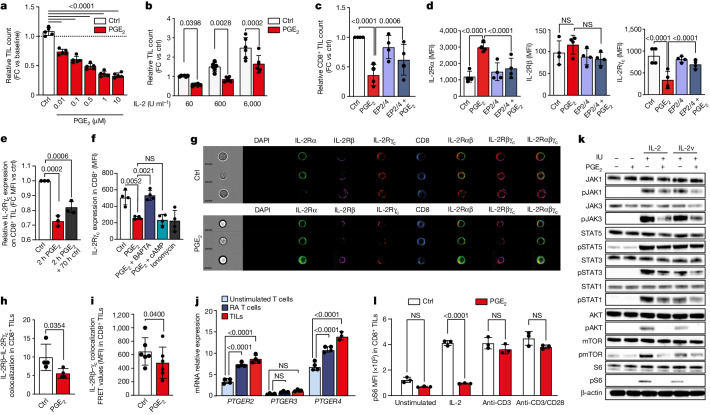
PGE_2_–EP2/EP4 signalling restricts IL-2 signalling in TILs by deregulating the IL-2R complex. **a**, Relative TIL count following treatment with PGE_2_ at various doses for 5 days (*n* = 5). FC, fold change. **b**, Relative TIL count following treatment for 72 h with PGE_2_ at different doses of IL-2 (*n* = 6). Ctrl, control. **c**, Relative CD8^+^ TIL count following treatment for 72 h with PGE_2_, EP2/EP4 antagonists (EP2/4), or combined treatment (*n* = 4). **d**, Surface expression of IL-2Rα, IL-2Rβ and IL-2Rγ_c_ in CD8^+^ TILs treated with PGE_2_ and EP2/EP4 antagonists for 72 h (*n* = 4). MFI, mean fluorescence intensity. **e**, Relative IL-2Rγ_c_ expression in CD8^+^ TILs treated with PGE_2_ for 2 h, or treated with PGE_2_ for 2 h and then re-exposed to medium without PGE_2_ for 70 h (*n* = 3). **f**, IL-2Rγc expression in unstimulated T cells treated with PGE_2_, the calcium chelator BAPTA, the cAMP antagonist Rp-8-CPT, ionomycin or combined treatment for 2 h (*n* = 4). **g**,**h**, Flow cytometry image of IL-2Rα, IL-2Rβ and IL-2Rγ_c_ expression (**g**; representative of four biological replicates) and colocalization of IL-2Rβ and IL-2Rγ_c_ in CD8^+^ TILs (**h**) upon 24 h treatment with PGE_2_, assessed by ImageStream (*n* = 4). A 7 μm scale bar is shown at bottom left of each row. **i**, FRET analysis of IL-2Rβγ_c_ in TILs treated with PGE_2_ for 24 h (*n* = 6). **j,** Relative mRNA expression of indicated genes in unstimulated T cells, RA T cells and TILs (*n* = 4). **k**, IL-2 signalling in RA T cells treated with PGE_2_ for 48 h and subsequently stimulated with IL-2 or IL-2v for 15 min (representative of 3 biological replicates). **l**, pS6 levels in CD8^+^ TILs treated for 2 h with PGE_2_ and subsequently stimulated for 30 min with IL-2, anti-CD3 or anti-CD3 plus anti-CD28 (anti-CD3/CD28) (*n* = 3). Data are mean ± s.d. Paired two-tailed *t*-test (**h**,**i**); one-way ANOVA with Dunnett’s post hoc test for multiple comparisons (**a**–**f**,**j**,**l**). Independent biological samples were used; exact numbers of biological replicates are listed in each panel. pJAK3, pS6, pAKT, STAT1, STAT3, pSTAT3, JAK1, pJAK1 and STAT5 were run on separate gels for blotting.

**Extended Data Fig. 3:**
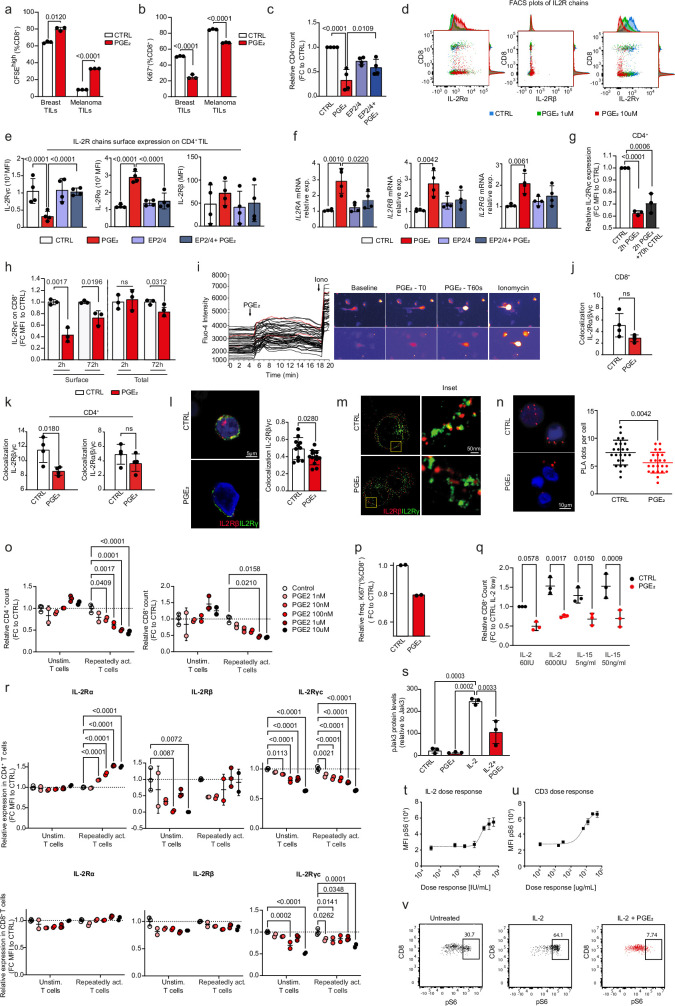
PGE_2_-EP2/EP4 signalling restricts IL-2 mediated TIL proliferation by deregulating IL-2R complexes. **a**, Frequency of CFSE^high^ and **b**, Ki67^+^TILs (% CD8^+^) treated with PGE_2_ for 5d in high dose IL-2 (6000IU/ml) (n = 3). **c**, Relative CD4^+^ count (Fold change to CTRL) of TILs treated for 72 h with PGE_2_ +/− EP2/EP4 antagonists (n = 4). **d**, Representative flow cytometry plots of IL-2Rα/β/γ_c_ chains surface expression on CD8^+^ TILs treated or not with PGE_2_ at 1 or 5uM. **e**, IL-2Rγ_c_, IL-2Rα and IL-2Rβ surface expression in CD4^+^ TILs treated with PGE_2_ +/− EP2/EP4 antagonists for 72 hrs (n = 4). **f**, Relative mRNA expression (Fold change to CTRL) of *IL-2RA*, *IL-2RB* and *IL-2RG* in TILs treated with PGE_2_ +/− EP2/EP4 antagonists (n = 4). **g**, Relative IL-2Rγ_c_ expression (Fold change to CTRL) in CD4^+^ TILs treated with PGE_2_ for 2 h, or for 2h and then re-exposed to media without PGE_2_ for 70 h (n = 3). **h**, Relative surface and total IL-2Rγ_c_ expression on CD8^+^ TILs (Fold change to CTRL) after 2 h or 72 h PGE_2_ treatment (n = 3). **i**, Time-lapse assessment (left) and representative image (right) of calcium mobilization in TILs upon PGE_2_ (representative from 2 biological replicates). **j**, Colocalization of IL-2Rα/β/γ_c_ chains by ImageStream in CD8^+^ and **k**, CD4^+^ TILs treated with PGE_2_(n = 4). **l**, Colocalization of IL-2Rβ and IL-2Rγ_c_ by confocal microscopy in TILs treated with 24 h PGE_2_ (representative from 4 biological replicates). **m**, Representative dSTORM image of IL-2Rβ and IL-2Rγ_c_ levels in TILs treated with 24 h PGE_2_ (representative of 2 biological replicates). **n**, Proximity ligation assay (PLA) images and quantification of IL-2Rβ/γ_c_ in TILs upon 24 h PGE_2_ (representative of 3 biological replicates). **o**, Relative cell count (Fold change to CTRL) of unstimulated or repeatedly activated **left)** CD4^+^ and **right)** CD8^+^ T cells upon 72 h PGE_2_ at different doses (n = 3). **p**, Relative frequency (Fold change to CTRL) of Ki67^+^(%CD8^+^) repeatedly activated T cells after PGE_2_ for 5d (n = 2). **q**, Relative cell count (Fold change to CTRL IL-2 low) of repeatedly activated CD8^+^ T cells treated with PGE_2_ in the presence of IL-2 or IL-15 for 48 h (n = 3). **r**, Relative IL-2Rα, IL-2Rβ and IL-2Rγ_c_ expression in unstimulated and repeatedly activated **top)** CD4^+^ and **bottom)** CD8^+^ T cells upon increasing doses of PGE_2_ (Fold change to CTRL) (n = 3). **s**, Densitometry quantification of pJAK3 levels in repeatedly activated T cells from experiment in Fig. 2k (n = 3). Samples derived from the same experiments and gels/blots were processed in parallel. **t**, pS6 dose response curves in TILs after 30 min stimulation with IL-2 or **u**, anti-CD3 (n = 3). **v**, Representative flow cytometry plots of pS6 expression in TILs stimulated with IL-2 or IL-2 and PGE_2_. Data are presented as the mean ± S.D. Statistical comparisons were performed using paired (j,k) or unpaired two-tailed t test (l,n,p) or one-way ANOVA (a,b,c,e,f,g,h,o,q,r,s) with Dunnett post-hoc test for multiple comparisons. Independent biological samples were used with exact numbers of biological replicates listed in each panel. p ≥ 0.05, not significant (ns).

PGE_2_ directly affects the cytotoxic function of T cells^13^. However, how PGE_2_ inhibits IL-2 dependent TIL expansion remains unknown. We found that exogenously added PGE_2_ attenuated the trophic effect of IL-2 on human TILs ex vivo in a dose-dependent manner (Fig. 2a) and suppressed TIL proliferation in culture at all concentrations of IL-2, including at the highest concentrations used in pre-REP (Fig. 2b and Extended Data Fig. 3a,b). Moreover, blockade of the PGE_2_ receptors EP2 and EP4 (EP2/EP4) with small molecule antagonists abrogated the suppressive effect of PGE_2_ on human TIL expansion from tumour tissue in response to IL-2 ex vivo (Fig. 2c and Extended Data Fig. 3c). Thus, PGE_2_ is directly responsible for limiting the IL-2 dependent expansion in human TILs via EP2 and EP4 signalling, similar to observations in a mouse model reported in the accompanying Article^14^.

We next assessed how PGE_2_ affects IL-2 sensing in human TILs. The IL-2R complex comprises three chains, IL-2Rα (also known as CD25), IL-2Rβ (also known as CD122) and the IL-2R common γ-chain^15^ IL-2Rγ_c_ (also known as CD132). Exposure to PGE_2_ reduced surface expression of IL-2Rγ_c_ protein in CD8^+^ TILs and CD4^+^ TILs, whereas EP2/EP4 antagonists abrogated this loss (Fig. 2d and Extended Data Fig. 3d,e). *IL2RG* mRNA was upregulated after 72 h of PGE_2_ exposure, indicating that the loss of surface IL-2Rγ_c_ was caused by post-transcriptional regulation (Extended Data Fig. 3f). Of note, the effect of PGE_2_ in reducing surface expression was selective for IL-2Rγ_c_, as both mRNA and surface protein levels of IL-2Rα increased significantly under the same conditions, whereas we observed an increase in mRNA but no changes at protein level for IL-2Rβ (Fig. 2d and Extended Data Fig. 3d–f). Short exposure of TILs to PGE_2_ (2 h) was sufficient to produce sustained loss of surface IL-2Rγ_c_ for 72 h, even when cells were returned to PGE_2_-free medium after 2 h, indicating that even transient exposure to PGE_2_ in tumour tissue can be detrimental to TILs (Fig. 2e and Extended Data Fig. 3g). Conversely, downregulation of the total cell IL-2Rγ_c_ protein content (including intracellular protein) reached significance only after 72 h of exposure (Extended Data Fig. 3h).

Calcium signalling can cause the degradation of IL-2Rγ_c_ protein in natural killer cells^16^ and can function as a second messenger alongside cAMP downstream of the PGE_2_ receptors EP2/EP4^17,18^. We therefore reasoned that calcium signalling might be involved in the rapid loss of surface IL-2Rγ_c_ caused by PGE_2_ in human TILs. We found that PGE_2_ increased intracellular Ca^2+^ levels in human TILs, and that increasing intracellular Ca^2+^ in T cells through stimulation with the ionophore ionomycin directly induced loss of surface IL-2Rγ_c,_ mimicking PGE_2_ (Fig. 2f and Extended Data Fig. 3i). Consistently, the calcium chelator BAPTA—but not the cAMP antagonist Rp-8-CPT—prevented the PGE_2_-mediated downregulation of IL-2Rγ_c_ from the cell surface (Fig. 2f). Thus, PGE_2_ causes rapid loss of surface IL-2Rγ_c_ via Ca^2+^ flux.

We investigated whether the loss of IL-2Rγ_c_ upon PGE_2_ stimulation affects the assembly of IL-2Rβ–IL-2Rγ_c_ (IL-2Rβγ_c_) complexes, which are required for IL-2 signalling. Exposure to PGE_2_ reduced the surface colocalization of IL-2Rβ and IL-2Rγ_c_ in CD8^+^ and CD4^+^ TILs, as visualized and quantified by imaging flow cytometry (Fig. 2g,h and Extended Data Fig. 3j,k). Analysis of TILs exposed to PGE_2_ by confocal microscopy (Extended Data Fig. 3l) and by direct stochastic optical reconstruction microscopy (dSTORM) super-resolution microscopy (Extended Data Fig. 3m) showed a reduction in IL-2Rβ–IL-2Rγ_c_ surface colocalization, which was confirmed by fluorescence resonance energy transfer (FRET) (Fig. 2i) and proximity ligation assay (PLA) (Extended Data Fig. 3n). Thus, PGE_2_-mediated loss of IL-2Rγ_c_ protein expression in TILs impairs the assembly of IL-2Rβγ_c_ complexes in the plasma membrane.

To further dissect how the PGE_2_-induced loss of IL-2Rβγ_c_ surface heterodimers affects IL-2 signalling in antigen-experienced T cells, we took advantage of the RA T cell model (Extended Data Fig. 2). Similar to TILs, human RA T cells showed high expression of *PTGER2* (which encodes EP2) and *PTGER4* (which encodes EP4) (Fig. 2j) and, in contrast to unstimulated T cells, were highly susceptible to PGE_2_-mediated impairment of IL-2 dependent expansion (Extended Data Fig. 3o). Furthermore, the restricted proliferation under IL-2 induced by PGE_2_ in RA T cells (Extended Data Fig. 3p) could not be rescued adequately by addition of high concentrations of IL-2 or IL-15 (another cytokine that requires IL-2Rγ_c_) (Extended Data Fig. 3q). Of note, PGE_2_ signalling in RA T cells resulted in selective loss of IL-2Rγ_c_ protein expression on the cell surface (Extended Data Fig. 3r), again phenocopying TILs from patient tumour tissue.

Consequently, stimulation of PGE_2_-exposed RA T cells with exogenous IL-2 did not induce key signalling events downstream of IL-2Rγ_c_, including phosphorylation of JAK1, STAT1, STAT3 and JAK3 (Fig. 2k and Extended Data Fig. 3s) (JAK3 is known to be preferentially associated with the IL-2Rγ_c_^19^). Similarly, PGE_2_-induced unresponsiveness was also observed towards an IL-2 variant (IL-2v) that selectively binds to IL-2Rβγ_c_ (Fig. 2k). Notably, despite no observed decrease in STAT5 phosphorylation, PGE_2_ inhibited phosphorylation of AKT, mTOR and S6 in response to both IL-2 and IL-2v (Fig. 2k), indicating loss of mTOR signalling. In pre-REP, TILs resident in tumour fragments receive—in addition to IL-2—signals through the T cell receptor (TCR) and possibly through co-stimulatory CD28 from adjacent tumour-resident antigen-presenting cells^11^. To determine which receptor pathway is disrupted by PGE_2_ to drive loss of mTOR in TILs, we tested the effect of PGE_2_ on mTOR activation (via the phosphorylated (p)S6 response) in the context of stimulation through the TCR only (using anti-CD3), the TCR and the CD28 coreceptor, or IL-2 (Extended Data Fig. 3t–v). Notably, PGE_2_ impaired pS6 induction by IL-2, but did not affect the pS6 response to anti-CD3 or combined anti-CD3 and anti-CD28 (Fig. 2l). Together, these findings indicate that intratumoral PGE_2_ specifically compromises IL-2 responsiveness, thereby impairing AKT–mTOR–S6 signalling in human TILs.

## PGE_2_ mediates metabolic rewiring in TILs

**Fig. 3:**
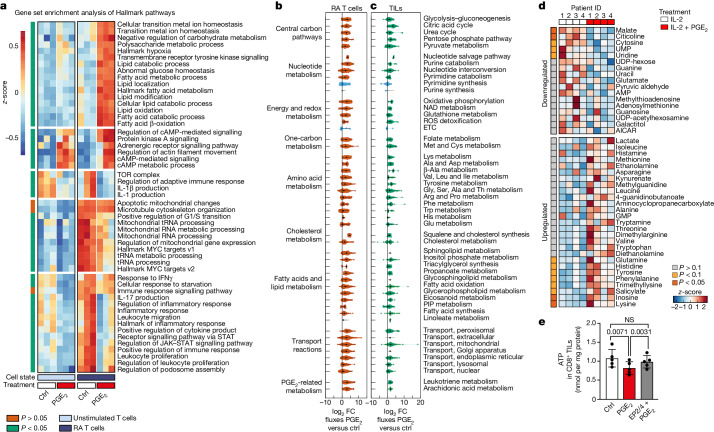
PGE_2_ rewires metabolism of TILs. **a**, Heat map of normalized expression, *z*-scored by row, of the top differentially expressed Hallmark signatures (*P* < 0.05) between unstimulated and RA CD8^+^ T cells treated with PGE_2_ for 24 h or untreated. *P* values (left column) indicate significance of differences between control and PGE_2_-treated RA CD8^+^ T cells in three patients (*n* = 3). *P* values (false discovery rate, Bonferroni-corrected) were calculated by applying GSEA on the average expression per group. **b**,**c**, Violin plot representation of fold changes in reaction rates of the inferred metabolic states for RA CD8^+^ T cells (**b**; *n* = 3) and CD8^+^ TILs (**c**; *n* = 1) upon 24 h exposure to PGE_2_. ETC, electron transport chain; PIP, phosphoinositide. **d**, Heat map representation of polar metabolites in CD8^+^ TILs upon PGE_2_ treatment (*n* = 4). *P* values (left column) were calculated using two-tailed paired *t*-test for the peak areas of the corresponding metabolites. **e**, ATP quantification by ELISA in CD8^+^ TILs treated for 24 h with PGE_2_, EP2/EP4 antagonists or combined treatment (*n* = 5). Data are mean ± s.d. One-way ANOVA with Dunnett’s post hoc test for multiple comparisons (**e**). Independent biological samples were used; exact numbers of biological replicates are listed in each panel.

**Extended Data Fig. 4:**
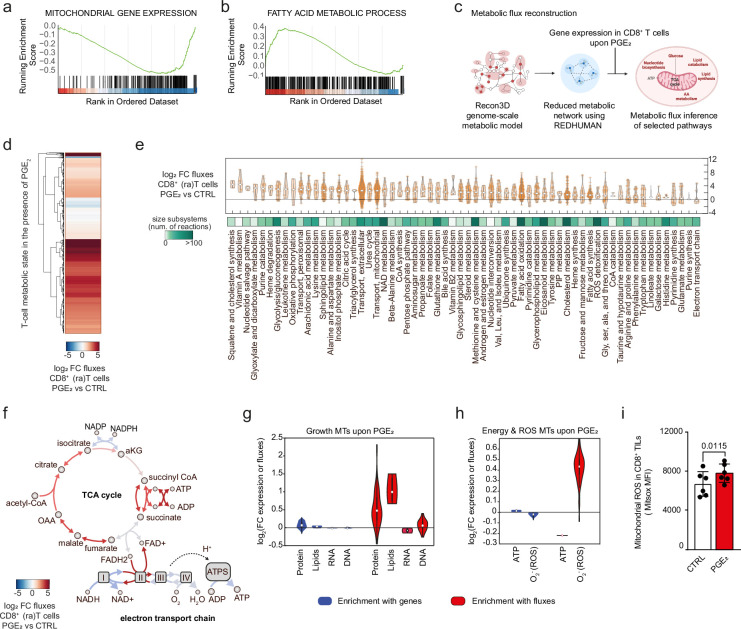
PGE_2_ rewires metabolism of TILs. **a-b**, Gene set enrichment analysis (GSEA) enrichment plot for **a**, mitochondrial gene expression and **b**, fatty acid metabolic process in repeatedly activated CD8^+^ T cells after 24 h of PGE_2_. The y-axis represents enrichment score and the x-axis shows genes (vertical black lines) represented in the pathways. The coloured band at the bottom represents the degree of correlation of the expression of these genes (red for a high gene expression and blue for a low gene expression). **c**, Schematic representation of the systems biology approach used to reconstruct metabolic models and infer fluxes and metabolic states consistent with the gene expression profile of repeatedly activated CD8^+^ T cells and TILs treated with PGE_2_ for 24 h (created with BioRender.com). **d**, Heatmap representation of deregulation of metabolic fluxes (row) inferred from the generated metabolic model upon PGE_2_ treatment in repeatedly activated CD8^+^ T cells (n = 3). **e**, Violin plot representation of reaction rate fold changes associated by metabolic subsystems upon PGE_2_ treatment in repeatedly activated CD8^+^ T cells (n = 3). **f**, Representative map of tricarboxylic acid cycle and electron transport chain (ETC) reactions, coloured by flux deregulation (blue for downregulated and red for upregulated inferred state). **g-h**, Violin plot representation of metabolic task (MT) derived for **g**, cell growth and **h**, energy and reactive oxygen species enrichment analysis using gene expression data (blue) or the inferred fluxes (red) in repeatedly activated CD8^+^ T cells upon PGE_2_ treatment (n = 3). **i**, Mitochondrial ROS levels in CD8^+^ TILs in response to PGE_2_ (n = 6). Data are presented as the mean ± S.D. Statistical comparisons were performed using paired two-tailed t test. Independent biological samples were used with exact numbers of biological replicates listed in each panel.

We performed transcriptional analysis by bulk RNA-seq of unstimulated and RA CD8^+^ T cells after exposure to PGE_2_ (Fig. 3a and Supplementary Table 2). By analysis of Hallmark pathways, we found that both cell states showed increased expression of genes associated with protein kinase A signalling, cAMP-dependent signalling and metabolic processes (Fig. 3a) including the cAMP-dependent transcription factor genes *CREM* and *CREB3L3*^20^ (Extended Data Fig. 2e,f), indicative of ongoing EP2/EP4 signalling and consistent with previous reports^18,21^. However, specifically in RA CD8^+^ T cells, PGE_2_ suppressed multiple IL-2-mediated pathways associated with T cell proliferation and regulation of metabolism, including JAK–STAT signalling, leukocyte proliferation and the mTOR pathway (Fig. 3a). Moreover, gene set enrichment analysis (GSEA) revealed that PGE_2_ induced specific transcriptional changes in RA CD8^+^ T cells related to mitochondrial and lipid metabolism (Fig. 3a and Extended Data Fig. 4a,b). In line with these data, PGE_2_ exposure upregulated genes associated with dysfunctional exhaustion (for example, *ENPDT1* and *CTLA4*^22^), cell cycle arrest (*CABLES1*^23^), mTOR inhibition (*DDIT4*^24^), lipid metabolism (*EPAS1* (also known as *HIF2a*)) and ferroptosis (*SLC47A1*^25^), a type of cell death caused by unrestricted lipid peroxidation (Extended Data Fig. 2e,f). This suggested that PGE_2_ produces important metabolic effects in RA T cells, which could affect expansion and survival of tumour-reactive TILs.

To learn more, we reconstructed a computational model to infer metabolic fluxes compatible with the gene expression profiles induced by PGE_2_ in RA T cells^26^ (Extended Data Fig. 4c and Methods). A general activation of metabolic reaction fluxes in response to PGE_2_ was inferred, including the central carbon pathway and amino acid and lipid metabolism (Fig. 3b and Extended Data Fig. 4d,e), implying that PGE_2_ imposed additional metabolic tasks on RA CD8^+^ T cells. An upregulation of glutathione metabolism and the reactive oxygen species (ROS) detoxification pathway was noted, suggesting increased oxidative stress imposed by PGE_2_. Conversely, nucleotide synthesis and the electron transport chain were suppressed (Fig. 3b, Extended Data Fig. 4e,f). Notably, and similarly to RA CD8^+^ T cells, PGE_2_ induced the downregulation of purine and pyrimidine synthesis and suppressed the electron transport chain in human CD8^+^ TILs (Fig. 3c Supplementary Table 3). Together, these data suggested that severe mitochondrial dysfunction and lack of nucleotide synthesis are at the basis of PGE_2_-mediated suppression of human CD8^+^ TIL expansion.

We used the RA CD8^+^ T cell model to further infer how T cells might utilize metabolic pathways to perform key functions such as production of macromolecules needed for growth or proliferation (proteins, lipids, DNA and RNA), energy production (ATP) or stress response (superoxide anion and ROS) upon PGE_2_ exposure^27^. We found a downregulation of RNA synthesis, concomitant with an upregulation of protein and lipid production, including complex lipid synthesis and fatty acid oxidation (Extended Data Fig. 4g). Of note, ATP-producing pathways exhibited less flux following exposure to PGE_2_, and cells were inferred to produce more ROS (Extended Data Fig. 4h).

To determine the relevance of these computational findings, we performed mass spectrometry-based targeted metabolomics analysis of expanded human CD8^+^ TILs exposed to PGE_2_ (Fig. 3d and Supplementary Table 4). We evaluated nucleotides, nucleosides, intermediates of the tricarboxylic acid cycle and free fatty acids (FFAs). Consistent with the above flux reconstruction, PGE_2_ induced a significant decrease in uridine nucleoside as well as nucleotides such as guanine, cytosine, uridine monophosphate (Fig. 3d). Moreover, we found a significant downregulation of malate, suggesting that PGE_2_ downregulates the malate–aspartate shuttle in human TILs (Fig. 3d), which is essential for ATP synthesis^28^. Finally, we confirmed a significant decrease in ATP production (Fig. 3e) and increased ROS levels (Extended Data Fig. 4i) upon PGE_2_ exposure in human CD8^+^ TILs. Collectively, these data indicate that PGE_2_ rewires the metabolism of human TILs, boosting lipid metabolism and increasing ROS production while deregulating energy and nucleotide synthesis, overall pointing towards mitochondrial dysfunction.

## PGE_2_ drives oxidative stress in TILs

**Fig. 4:**
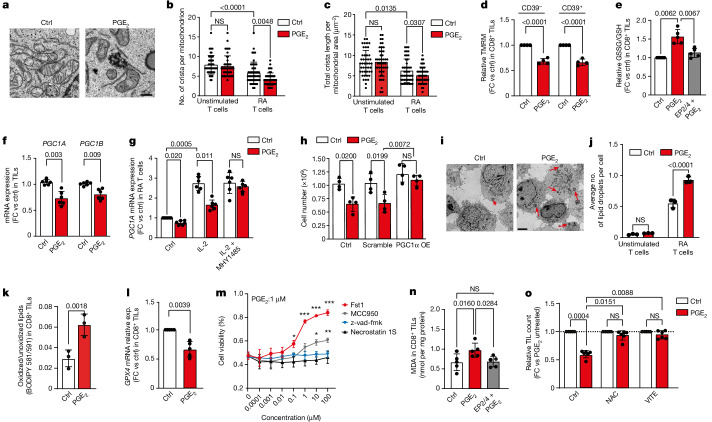
PGE_2_ increases oxidative stress in TILs by impairing the IL-2–mTOR–PGC1α axis, leading to ferroptosis. **a**, Representative electron microscopy images of RA T cells with and without 24 h PGE_2 _treatment (*n* = 3). Scale bar, 500 nm. **b**,**c**, Representative cristae number and length per mitochondrion in unstimulated and RA T cells upon 24 h PGE_2_ treatment (*n* = 3). **d**, Fold change (relative to control) of mitochondrial potential (indicated by tetramethylrhodamine methyl ester (TMRM)) in CD39^−^ and CD39^+^ CD8^+^ TILs after 24 h PGE_2_ treatment (*n* = 4). **e**, Fold change (relative to control) of oxidized/reduced glutathione quantified by ELISA in CD8^+^ TILs upon 24 h PGE_2_ treatment with or without EP2/EP4 antagonists (*n* = 4). **f**, Relative *PGC1A* and *PGC1B* mRNA expression in TILs after 48 h PGE_2_ (*n* = 6). **g**, Relative *PGC1A* mRNA expression in RA T cells treated with 12 h PGE_2_ with or without an mTOR activator (MHY1485) and subsequently stimulated with IL-2 for 15 min (*n* = 6). **h**, Cell count of RA OT-1 mouse T cells overexpressing PGC1α (PGC1α OE) upon 72 h PGE_2_ exposure (*n* = 4). **i**,**j**, Electron microscopy images (**i**; representative of three biological replicates) and mean number of lipid droplets per cell in unstimulated T cells and RA T cells upon 24 h PGE_2_ exposure (*n* = 3). Scale bar, 3 μm. **k**, Lipid peroxidation in CD8^+^ TILs after 48 h PGE_2_ exposure (*n* = 3). **l**, Fold change in *GPX4* mRNA expression (exp.) in CD8^+^ TILs after 48 h PGE_2_ (*n* = 5). **m**, Frequency of viable TILs after 72 h treatment with PGE_2_ and indicated concentrations of Fst1 (ferroptosis inhibitor), MCC905 (pyroptosis inhibitor), z-vad-fmk (apoptosis inhibitor) or necrostatin 1S (necroptosis inhibitor) (*n* = 3). Two-way ANOVA with Dunnett’s post test; *P < 0.05, **P < 0.01, ***P < 0.001. **n**, MDA quantification in CD8^+^ TILs upon 48 h PGE_2_ exposure with or without EP2/EP4 antagonists, using ELISA (*n* = 5). **o**, Relative cell count of TILs upon 72 h PGE_2_ exposure with or without NAC or vitamin E (VITE) (*n* = 6). Data are mean ± s.d. Paired two-tailed *t*-test (**k**,**l**); one-way ANOVA with Dunnett’s post hoc test for multiple comparisons (**b**–**h**,**j**,**n**,**o**). Independent biological samples were used; exact numbers of biological replicates are listed in each panel.

**Extended Data Fig. 5:**
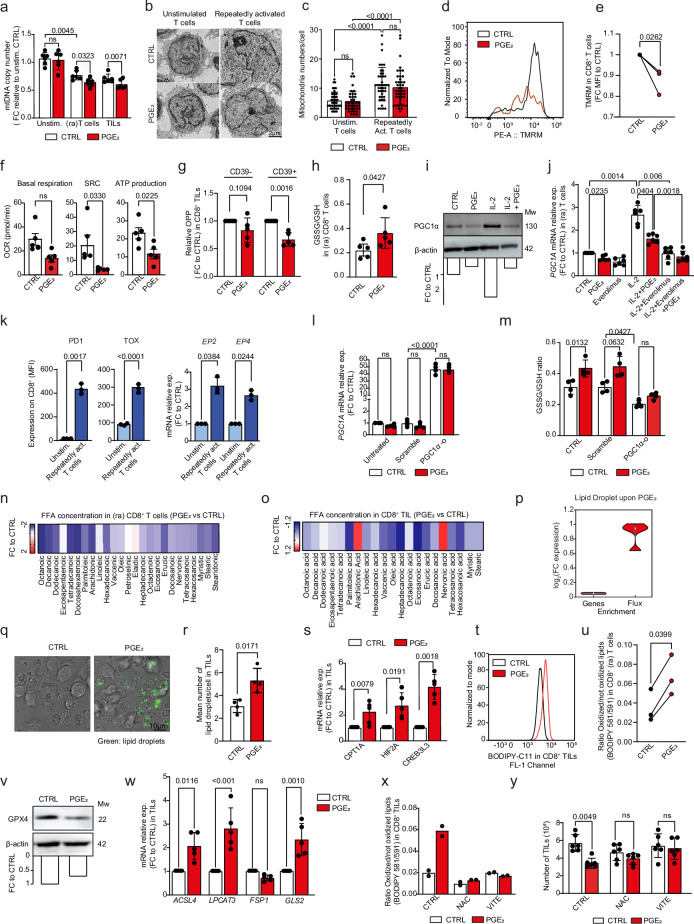
PGE_2_ affects mitochondrial fitness, T cell oxidative response and lipid metabolism. **a**, Relative mitochondrial DNA copy number in unstimulated, repeatedly activated T cells and TILs after 24 h PGE_2_ (Fold change to CTRL) (n = 6). **b**, Representative Electron Microscopy images and **c**, representative quantitative plot of mitochondrion number/cell in unstimulated and repeatedly activated T cells upon 24 h PGE_2_ (n = 3). **d**, Representative histogram of mitochondrial potential (TMRM) staining in CTRL versus PGE_2_ treated TILs. **e**, Fold change (relative to CTRL) of mitochondrial potential (TMRM) in repeatedly activated CD8^+^ T cells after 24 h PGE_2_ (n = 3). **f**, Quantification of basal respiration, spare respiratory capacity (SRC) and ATP production in repeatedly activated T cells treated with PGE_2_ for 24 h (n = 5). **g**, Fold change (relative to CTRL) of protein synthesis (OPP) in CD39^−^ and CD39^+^ CD8^+^ TILs treated with PGE_2_ for 24 h (n = 5). **h**, Oxidized (GSSG)/reduced (GSH) glutathione ratio quantified by mass-spectromety in CD8^+^ repeatedly activated T cells treated with PGE_2_ for 24 h (n = 5). **i**, Western Blot (top) and quantification (bottom) of PGC1α in TILs treated with IL-2 and PGE_2_ for 48 h (representative of 2 biological replicates). **j**, *PGC1*A mRNA relative expression in repeatedly activated T cells treated with PGE_2_ or mTOR inhibitor Everolimus for 12 h and then stimulated with IL-2 for 15 min (n = 6). **k**, PD1, TOX protein expression and *EP2* and *EP4* gene expression in unstimulated or repeatedly activated murine OT1 T cells (n = 3). **l**, *PGC1*A mRNA relative expression in repeatedly activated OTI murine T cells transduced with a PGC1α overexpressing vector (n = 4). **m**, Oxidized (GSSG)/reduced (GSH) glutathione ratio in response to 72 h PGE_2_ in repeatedly activated OTI murine T cells transduced with a PGC1α -overexpressing vector (n = 4). **n**, Heatmap of free fatty acids relative abundance (%CTRL) measured by mass spectrometry in repeatedly activated CD8^+^ T cells (n = 5) and in **o**, CD8^+^ TILs treated with PGE_2_ for 24 h (n = 4). **p**, Violin Plot representation (Fold change to CTRL) of lipid droplets metabolic task in repeatedly activated CD8^+^ T cells treated with PGE_2_ (n = 3). **q**, Microscopy image (representative of 4 biological replicates) and **r**, mean lipid droplets/cells in TILs upon 24 h PGE_2_ (n = 4). **s**, mRNA relative expression of *CPT1A*, *HIF2α*, *CREB3L3* in TILs treated with PGE_2_ for 48 h (n = 5). **t**, Representative histogram of BODIPY-C11 lipid peroxidation staining in CTRL versus PGE_2_ treated CD8^+^ TILs. **u**, Lipid peroxidation quantification in repeatedly activated CD8^+^ T cells treated with PGE_2_ for 48 h (n = 3). **v**, Western blots of GPX4 protein expression in TILs treated or not with PGE_2_ (representative of 2 biological replicates). **w**, mRNA relative expression of *ACSL4*, *LPCAT3*, *FSP1* and *GLS2* in TILs treated with PGE_2_ for 48 h (n = 5). **x**, Lipid peroxidation quantification (n = 2) and **y**, Relative cell count (Fold change to CTRL) of TILs treated with PGE_2_ +/− NAC or vitamin E (VITE) for 72 h (n = 6). Data are presented as mean ± S.D. Statistical comparisons were performed using paired (f,h,r,u) or unpaired two-tailed t test (e,k) or one-way ANOVA (a,c,g,j,l,m,s,w,y) with Dunnett post-hoc test for multiple comparisons. Results were pooled from 3 experiments with n = 5 mice per group in each experiment (l,m). Independent biological samples were used with exact numbers of biological replicates listed in each panel. p ≥ 0.05, not significant (ns).

We next evaluated whether PGE_2_ deregulates mitochondrial function in RA CD8^+^ T cells and TILs. Notably, both TILs and RA T cells exhibited decreased copy number of mitochondrial DNA (mtDNA) relative to unstimulated donor T cells, whereas mtDNA was further reduced in both cell types by PGE_2_ exposure (Extended Data Fig. 5a). Moreover, ultrastructural analysis of mitochondria in RA T cells revealed—similar to previous reports for human TILs^29^—reduced numbers and length of cristae per mitochondrion but an increased total number of mitochondria per cell relative to unstimulated T cells (Fig. 4a–c and Extended Data Fig. 5b,c). Furthermore, exposure of RA CD8^+^ T cells to PGE_2_ reduced the mitochondrial membrane potential (Δ*Ψ*_m_) (Extended Data Fig. 5d,e), an indicator of mitochondrial function^30^, in addition to mitochondrial respiration and ATP production (Extended Data Fig. 5f). Similarly, PGE_2_ decreased Δ*Ψ*_m_ in both terminally differentiated CD39^+^ and less differentiated CD39^−^ TILs (Fig. 4d). However, protein translation—quantified via *O*-propargyl-puromycin (OPP) levels—which correlates with energy production and mTOR–S6 signalling^31^, was more compromised in the CD39^+^ population (Extended Data Fig. 5g). Finally, because of the importance of mitochondria for cellular oxidative homeostasis, we determined the ratio of oxidized glutathione (GSSG) to the reduced form (GSH). Exposure to PGE_2_ increased the GSSG/GSH ratio both in CD8^+^ TILs (Fig. 4e) and in RA CD8^+^ T cells (Extended Data Fig. 5h), revealing an oxidative imbalance upon exposure to PGE_2_.

mTOR signalling has a key role in mitochondrial function. Given the profound suppression of mTOR by PGE_2_, we evaluated the expression of the mTOR target PGC1α, a transcription co-activator that coordinates mitochondrial biogenesis and antioxidant activity^32^. PGE_2_ exposure of human TILs reduced *PGC1A* and *PGC1B* gene expression (Fig. 4f) and prevented *PGC1A* upregulation in response to high-dose IL-2 in TILs (Extended Data Fig. 5i,j). The mTOR inhibitor everolimus hampered *PGC1A* upregulation (Extended Data Fig. 5j), mimicking PGE_2_, whereas the mTOR activator MHY1485 rescued IL-2-induced *PGC1A* expression when RA T cells were exposed to PGE_2_ (Fig. 4g), demonstrating that PGE_2_ drives transcriptional repression of *PGC1A* through suppression of mTOR signalling. To test whether loss of *PGC1A* drives the oxidative imbalance upon exposure to PGE_2_, we overexpressed PGC1α in CD8^+^ T cells. We used TCR transgenic mouse OT-1 T cells, which similar to human T cells, upregulated *PTGER2* and *PTGER4* along with PD-1 and TOX upon repeated TCR activation (Extended Data Fig. 5k). Overexpression of *PGC1A* rendered these cells resistant to PGE_2_ compared with control treated cells (Fig. 4h and Extended Data Fig. 5l). Of note, PGC1α-overexpressing OT-1 cells had a lower GSSG/GSH ratio upon PGE_2_ exposure in vitro (Extended Data Fig. 5m), indicating a rescue of oxidative imbalance. Together, these findings demonstrate that PGE_2_ drives mitochondrial dysfunction and aggravates oxidative stress in dysfunctional T cells by directly suppressing the IL-2–mTOR–PGC1α axis.

## PGE_2_ mediates TILs death via ferroptosis

Lipid metabolism is a key survival pathway when mTORC1 is inhibited and FFAs are used for energy production via fatty acid oxidation^33^. However, in the presence of impaired mitochondrial function and antioxidants capabilities, accumulation of lipid peroxides may lead to cell death via ferroptosis^34,35^. We therefore investigated whether PGE_2_, by shutting down mTOR signalling, reducing antioxidant competence and simultaneously increasing lipid utilization, creates conditions that are favourable for TIL ferroptosis. Using mass spectrometry, we found that several short and long-chain FFAs as well as several carnitine and acetyl carnitine species were depleted in PGE_2_-exposed RA CD8^+^ T cells and CD8^+^ TILs (Extended Data Fig. 5n,o). We reasoned that because synthesis of complex lipids was upregulated in the context of decreased T cell proliferation upon PGE_2_ exposure, CD8^+^ T cells stored FFAs in lipid droplets, which might protect them from lipotoxic damage to mitochondria^36^. By flux reconstruction, we inferred an upregulation by PGE_2_ of pathways leading to increased lipid droplet formation in RA CD8^+^ T cells (Extended Data Fig. 5p), which we readily detected in RA CD8^+^ T cells and TILs but not in unstimulated CD8^+^ T cells upon PGE_2_ treatment (Fig. 4i,j and Extended Data Fig. 5q,r). Similar to RA CD8^+^ T cells, we detected upregulated expression of lipid metabolism genes in PGE_2_-treated TILs, including *CREB3L3*, which has been implicated in fatty acid oxidation^37^; *CPT1A*, which encodes a transporter required for FFA transport across the mitochondrial inner membrane; and *HIF2a*, which encodes a transcription factor that modulates lipid metabolism and lipid droplet formation^38^ (Extended Data Fig. 5s). We also found that PGE_2_ induced lipid peroxidation in TILs and RA CD8^+^ T cells (Fig. 4k and Extended Data Fig. 5t,u).

To determine whether this PGE_2_-mediated deregulation of lipid metabolism and lipid peroxide accumulation in TILs drives ferroptosis, we evaluated expression levels of GPX4, a key enzyme of the glutathione system that protects cells from ferroptosis through the detoxification of lipid peroxides^39^. We found a significant reduction in *GPX4* mRNA and GPX4 protein levels in CD8^+^ TILs exposed to PGE_2_ (Fig. 4l and Extended Data Fig. 5v). In addition, PGE_2_ upregulated genes linked to the ferroptosis pathway, such as *ACSL4*, *LPCAT3* and *GLS2*, in TILs (Extended Data Fig. 5w). Of note, blockade of ferroptosis using the specific inhibitor ferrostatin (Fst1) resulted in a prominent rescue of TIL survival despite PGE_2_ exposure (Fig. 4m). This rescue was selective for Fst1-mediated ferroptosis inhibition, whereas inhibition of apoptosis (using z-VAD-FMK), necroptosis (using necrostatin 1S) or pyroptosis (MCC950) were ineffective in restoring TIL expansion (Fig. 4m). Collectively, these data suggest that PGE_2_-induced TIL death is mediated by ferroptosis. In line with this notion, TILs exposed to PGE_2_ exhibited an increase in the intracellular levels of malondialdehyde (MDA), a lipid peroxidation end-product associated with ferroptosis^40^ (Fig. 4n). Moreover, MDA accumulation was abrogated by pre-incubating TIL with EP2/EP4 inhibitors (Fig. 4n). Finally, exposure of TILs to the anti-oxidative compounds *N*-acetylcysteine (NAC) or vitamin E reduced peroxidized lipid accumulation in the presence of PGE_2_ (Extended Data Fig. 5x) and protected TILs from the suppressive effects of PGE_2_ (Fig. 4o and Extended Data Fig. 5y).

## PGE_2_ blockade improves TIL-ACT product

**Fig. 5:**
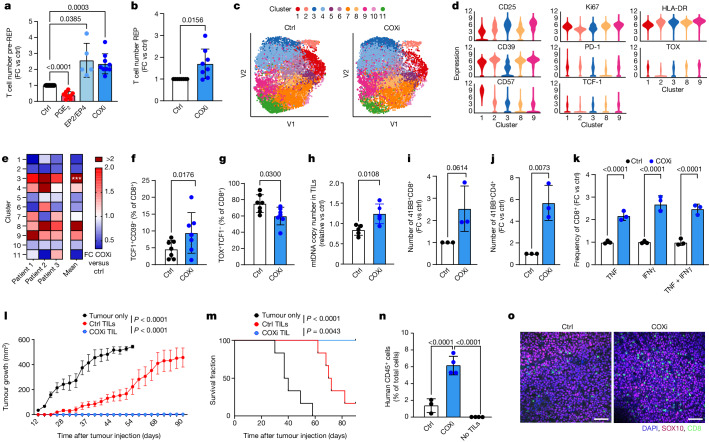
Blockade of the PGE_2_–EP2/EP4 axis increases TIL expansion, fitness and tumour reactivity. **a**, Relative number of pre-REP TILs from different solid tumours treated with IL-2, IL-2 plus PGE_2_, IL-2 plus EP2/EP4 antagonists or IL-2 plus ketorolac (COXi) during the first 48 h of culture (*n* = 13). **b**, Relative number of REP TILs in the COXi group from different solid tumours (*n* = 8). **c**, Uniform manifold approximation and projection (UMAP) projection of 34-parameter CyTOF data showing sub-clustering of control and COXi REP TILs from three patients with melanoma (*n* = 3). **d**, Violin plots showing expression of indicated proteins detected by CyTOF in five different clusters in control and COXi REP TILs. **e**, Fold change frequency of CD8^+^ REP TILs per cluster in control and COXi groups (*n* = 3). **f**,**g**, Frequency of TCF1^+^CD39^−^ (**f**) and TOX^+^TCF1^−^ (**g**) TILs as a percentage of CD8^+^ TILs in control and COXi REP TILs (*n* = 7). **h**, Fold change of mitochondrial DNA copy number in REP COXi TILs (*n* = 5). **i**,**j**, Relative cell count of tumour-reactive CD8^+^ (*n* = 3) (**i**) and CD4^+^ (**j**) TILs at the end of REP step (*n* = 3). Tumour reactivity was assessed via 41BB surface expression upon co-culture with autologous tumours. **k**, Fold change in frequency of TNF^+^, IFNγ^+^ or TNF^+^IFNγ^+^ CD8^+^ REP TILs as a percentage of CD8^+^ TILs upon overnight co-culture with autologous tumour cells (*n* = 3). **l**,**m**, Tumour growth kinetics (**l**) and survival curve (**m**) for mice treated with COXi and control REP TILs in a Winn assay transfer (*n* = 6 mice per group). **n**,**o**, Frequency as a percentage of total cells (**n**) and representative images (**o**) of intratumoral human CD45^+^ cells upon treatment of COXi or control REP TILs (*n* = 3 or 4 mice per group). Scale bars, 100 μm. Data are mean ± s.d. One-way ANOVA (a,e,k,n) or two-way ANOVA with Dunnett’s post hoc test for multiple comparisons (**l**,**m**); Cohen’s *D* test (**d**) (Supplementary Table 5); or paired (**f**–**h**) or unpaired (**b**,**i**,**j**) two-tailed *t*-test. Independent biological samples were used; exact numbers of biological replicates are listed in each panel.

**Extended Data Fig. 6:**
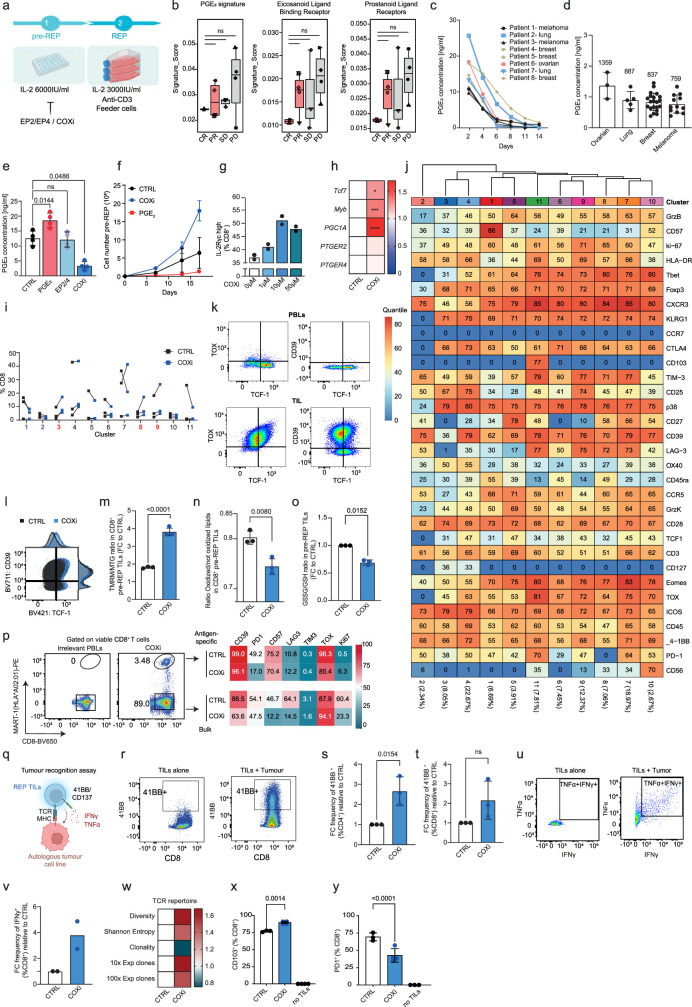
PGE_2_-EP2/EP4 axis blockade increases TIL expansion, fitness and tumour-reactivity. **a**, Schematic representation of conventional TIL expansion protocol. TILs are expanded from tumours fragments with IL-2 6000IU/ml for 14-28d (pre-REP phase) and then with IL-2 3000IU/ml, anti-CD3 and feeder cells for 14d (REP phase). **b**, Correlation between best overall clinical response at 3-months and “PGE_2_ signature”, “Eicosanoid ligand binding receptor” and “Prostanoid ligand receptors” signature scores in CD8^+^ TILs from REP-TIL product of melanoma patients enrolled in phase I ACT-TIL therapy trial (n = 13). CR: Complete Response, PR: Partial Response, SD: Stable Disease, PD: Progressive Disease. **c**, Time-course of PGE_2_ concentration (n = 8) and **d**, PGE_2_ concentration at day 7 in the supernatant of expanding pre-REP TILs derived from breast (n = 21), melanoma (n = 12), ovarian (n = 3), and lung (n = 5) tumours. **e**, Baseline PGE_2_ concentration in the supernatant of 48 h pre-REP cultures treated with PGE_2_, EP2/4 blockade or Ketorolac/COXi (n = 4). **f**, Kinetics of expansion of pre-REP TILs treated with IL-2 (6000IU/ml), IL-2 + PGE_2_, or IL-2 + Ketorolac/COXi at initiation of the culture (n = 2). **g**, Frequency of IL-2Rγ_c_^high^ CD8^+^ TILs (%CD8^+^) at day 7 of expansion in presence of different doses of Ketorolac/COXi (n = 2). **h**, Heatmap representation of *TCF7, Myb, PGC1A, PTGER2, PTGER4* mRNA expression in pre-REP TILs expanded with or without Ketorolac/COXi (n = 3). **i**, Frequency of CD8^+^ TILs per cluster between CTRL and Ketorolac/COXi expanded TILs from mass cytometry (n = 3). **j**, Heatmap representation of 32 CyTOF markers expression in each cluster. Frequency of CD8^+^ TILs per cluster are depicted at the bottom. **k**, Representative flow cytometry plots of TOX/TCF1 and TCF1/CD39 of CD8^+^ PBLs or TILs. **l**, Representative flow cytometry plot of TCF1/CD39 CTRL and COXi-CD8^+^TILs. **m**, Relative TMRM/mitotracker green ratio (Fold change to CTRL) (n = 3) and **n**, Lipid peroxidation quantification (n = 3) and **o**, Relative Oxidized (GSSG)/reduced (GSH) glutathione ratio (Fold change to CTRL) in pre-REP CD8^+^ Ketorolac/COXi TILs (n = 3). **p**, Representative flow cytometry plots and **p**, Phenotypic characterization of multimer stained MART-1 tumour-reactive CD8^+^ TILs expanded with IL-2 or IL-2+Ketorolac/COXi (n = 1). **q**, Schematic representation of autologous tumour cells and TILs co-culture assay. **r**, Representative flow cytometry plot of 41BB^+^ CD8^+^ TILs in absence (TILs alone) or presence (TILs + Tumour) of tumour cells. **s**, Relative frequency of tumour-reactive CD4^+^ TILs and **t**, CD8^+^ TILs at REP in the Ketorolac/COXi (Fold change to CTRL). Tumour-reactive T cells were assessed via 41BB surface staining expression upon co-culture with autologous tumour by flow cytometry staining (n = 3). **u**, Representative flow cytometry plot of TNFα^+^IFNγ^+^ CD8^+^ TILs in absence (TILs alone) or presence (TILs + Tumour) of tumour cells. **v**, Relative frequency (Fold change to CTRL) of tumour-reactive Ketorolac/COXi CD8^+^ TILs assessed by IFNγ^+^ expression upon co-culture with autologous tumour line in two out of the three melanoma patients tested (last patient had no IFNγ detected) (n = 2). **w**, TCRβ repertoire analysis of REP TILs expanded with IL-2 or IL-2+Ketorolac/COXi (n = 3). **x**, Frequency of CD103^+^ (n = 3) or **y**, PD1^+^ (%CD8^+^) intratumoural TILs in tumours from mice treated with CTRL or Ketorolac/COXi-expanded REP TILs (n = 3). Data are presented as the mean ± S.D. Statistical comparisons were performed using paired (n) unpaired two-tailed t test (m,o,s,t) or one-way ANOVA (b,e,h,x,y) with Dunnett post-hoc test for multiple comparisons. p ≥ 0.05, not significant (ns). Independent biological samples/patients were used with exact numbers of biological replicates listed in each panel. Panels a and p were created with BioRender.com.

Current pre-REP expansion protocols result in TILs being driven to proliferate from the tumour fragment in the presence of tumour-derived PGE_2_. We thus hypothesized that PGE_2_ blockade might restore IL-2 sensing and improve TIL expansion and tumour control (Extended Data Fig. 6a). We first analysed the TIL product of patients with melanoma undergoing TIL-ACT therapy and observed a correlation between high PGE_2_-related signature scores in CD8^+^ REP TILs and decreased clinical response^11,12^ (Extended Data Fig. 6b). Consistent with our analyses of melanoma tumour tissues (Fig. 1), we detected high levels of PGE_2_ across early pre-REP tumour fragment cultures from multiple tumour types (Extended Data Fig. 6c,d). Addition of exogenous PGE_2_ at the onset of culture restrained TIL expansion in response to IL-2 (Fig. 5a), whereas blocking EP2/EP4 receptors with specific antagonists, or blocking cyclooxygenases (COX) with the pan-COX inhibitor ketorolac (COXi), which efficiently reduced PGE_2_ in the culture medium (Extended Data Fig. 6e), significantly increased the number of expanded TILs during pre-REP (Fig. 5a and Extended Data Fig. 6f). When compared with standard IL-2-expanded TILs, TILs expanded from COXi-treated tumour fragments exhibited higher surface expression of IL-2Rγ_c_ (Extended Data Fig. 6g), predicting restored responsiveness to IL-2. Consistently, these cells exhibited higher expression of *PGC1A*, suggesting increased mitochondrial fitness, and enhanced expression of the transcription factor genes *TCF7* and *MYB* (Extended Data Fig. 6h), which regulate stem-like T cell longevity and proliferative competence^41^. Furthermore, pre-REP TILs cultured in the presence of COXi showed greater expansion during REP (Fig. 5b).

To further characterize the effect of blocking PGE_2_ in TIL cultures, we analysed control and COXi TIL products using 34-parameter mass cytometry by time of flight (CyTOF) (Supplementary Table 5). Unsupervised clustering analysis identified 11 clusters, 5 of which (clusters 1, 2, 3, 8 and 9) differed between control and COXi-treated TILs. Clusters 3, 8 and 9—enriched in the COXi group (Fig. 5c–e and Extended Data Fig. 6i,j)—were generally CD57^low^ and were characterized by either low CD39 and high IL-2Rα expression (cluster 3), high CD39 but low PD-1 expression (cluster 8), or high expression of the proliferation marker Ki67, IL-2Rα and TCF1 (cluster 9), indicative of more proliferation-competent precursor-like T cells. By contrast, clusters 1 and 2—enriched in control TILs—comprised IL-2Rα^low^CD57^hi^HLA-DR^low^PD-1^hi^TOX^high^ (cluster 1) or CD39^hi^Ki67^low^TCF1^low^ cells (cluster 2), characteristic of more terminally differentiated or dysfunctional effector T cells (Fig. 5c–e and Extended Data Fig. 6i,j).

Maintenance of more proliferation-competent precursor-like TILs upon addition of COXi was independently confirmed in seven patients with cancer by flow cytometry analysis, which demonstrated a relative increase in stem-like CD39^−^TCF1^+^ cells and a concomitant decrease in TOX^+^TCF1^−^ cells in the COXi group (Fig. 5f,g and Extended Data Fig. 6k,l).

In addition to the enhanced expansion potential and maintenance of precursor-like features (Fig. 5f,g), COXi TILs exhibited increased Δ*Ψ*_m_ (Extended Data Fig. 6m) and mitochondrial DNA content (Fig. 5h), and decreased peroxidised lipid levels and GSSG/GSH ratio (Extended Data Fig. 6n,o). These data confirm that attenuating PGE_2_ signals early during expansion improved TIL expansion and metabolic fitness.

We next addressed the key question of how tumour-reactive TILs are affected by COX blockade relative to bystander TILs. To capture tumour-reactive CD8^+^ T cells we tracked TILs recognizing the MART-1 (Melan-A_26–35*A27L_) peptide^42^. We observed increased Ki67 and decreased PD-1 and TOX expression levels selectively in MART-1-specific COXi TIL (Extended Data Fig. 6p), indicating that rescue from PGE_2_ suppression enhanced response to IL-2 selectively in tumour-reactive TILs. In line with these results, we found that addition of COXi in early pre-REP led to around 2.5-fold higher expansion of tumour-reactive TILs relative to conventional TILs, as measured by CD137 surface expression and IFNγ and TNF cytokine expression upon autologous in vitro tumour co-culture assay (Fig. 5i–k and Extended Data Fig. 6q–v). Moreover, these COXi TILs exhibited increased repertoire richness as well as decreased clonality and increased entropy (Extended Data Fig. 6w), indicating mobilization and maintenance of a broader T cell repertoire when PGE_2_ is attenuated.

We then determined whether the increased metabolic features and expansion potential of COXi TILs translated into better tumour control upon ACT. We co-administered control or COXi TILs subcutaneously with autologous patient-derived tumour cells into NSG mice. Unlike control TILs, which were unable over time to control tumour growth in this model, COXi TILs achieved complete tumour rejection (Fig. 5l,m and Supplementary Table 6). Consistent with these findings, following adoptive transfer into NSG mice bearing patient-derived xenograft melanoma tumours, COXi TILs exhibited markedly increased intratumoral abundance (Fig. 5n,o), along with higher expression of the integrin CD103 and lower expression of PD-1 (Extended Data Fig. 6x,y) compared with control TILs, pointing towards better persistence of qualitatively superior T cells. Together, these data suggest that blocking PGE_2_ during TIL manufacturing can enhance the performance of human TIL products upon ACT in vivo.

## Discussion

We currently lack sufficient understanding of the mechanisms that restrain functional TIL responses in the TME, limiting advance of cancer immunotherapies and TIL-ACT approaches. In this study and in the accompanying Article^14^, we show that PGE_2_ acts on cell-intrinsic features of mouse and human CD8^+^ TILs by negatively modulating IL-2 signalling, which critically restricts TIL proliferation and survival. Here, we show that the PGE_2_–EP2/EP4 axis rapidly downregulates surface IL-2Rγ_c_ and disrupts assembly of IL-2Rβγ_c_ dimers in the plasma membrane in CD8^+^ TILs. This phenomenon induced a state of IL-2 unresponsiveness or ‘anergy’, which collapses mTOR signalling and drives a metabolic rewiring of dysfunctional T cells, ultimately leading to mitochondrial impairment, irreparable oxidative stress, and death by ferroptosis.

Our findings identify the PGE_2_–EP2/EP4 signalling axis as a key mechanism underlying mitochondrial depolarization^28,43^ and deregulated metabolism of human CD8^+^ T cells in tumour tissue, and establish a mechanistic link to a defect in the IL-2–mTOR–pS6 pathway underlying inefficient responses by human TILs. Notably, these effects appear to be specific to mTOR but not to canonical STAT signalling. Of note, PGE_2_–EP2/EP4 signalling aggravated bioenergetic function of dysfunctional T cells such as RA T cells or tumour-reactive TILs. Tumour-reactive TILs inevitably acquire a dysfunctional phenotype when they repeatedly encounter antigen in the TME^44,45^ and thus might be particularly vulnerable to the deleterious effect of PGE_2_. Indeed, the PGE_2_–EP2/EP4 axis restricted antigen-specific T cell expansion in response to IL-2, and this effect was remarkably rapid and durable in vitro, suggesting that within the TME, cell niches with high local levels of PGE_2_ might swiftly engage and induce IL-2 anergy in approaching tumour-reactive cytotoxic CD8^+^ T cells, quickly collapsing their bioenergetics, thereby suppressing their expansion, and ultimately compromising the survival of these clonotypes. This phenomenon may also be exacerbated by limited IL-2 bioavailability in tumours, potentially due to PGE_2_-mediated induction of IL-2-scavenging regulatory T cells^46^.

Blocking the PGE_2_–EP2/EP4 axis early in pre-REP restored IL-2 signalling and maintained TILs with increased stem-like features and mitochondrial fitness, in line with their increased proliferative potential, resulting in expansion of TILs with a broadened TCR repertoire and increased tumour reactivity. All of these are key factors related to increased efficacy in TIL-ACT^1,8^. Our findings therefore reveal a ‘window of opportunity’ to intervene during TIL-ACT expansion by blocking the PGE_2_–EP2/EP4 axis or by protecting TILs with antioxidant drugs to resist the toxic influence of PGE_2_ within the TME. This would enable the amplification of IL-2 response and the rescue of tumour-reactive T cells, which are typically in a dysfunctional state and more susceptible to IL-2 deprivation.

In conclusion, our findings dissect the underlying mechanisms by which PGE_2_ inhibits IL-2 signalling and, as a result, human TIL proliferation, with important clinical implications for improving TIL-ACT and cancer immunotherapy.

## Methods

### Tumour samples

Tumour samples were collected from individuals with melanoma, non–small cell lung cancer, ovarian cancer, and breast cancer undergoing surgical treatment between October 2016 and August 2023 at the Centre Hospitalier Universitaire Vaudois (CHUV), Lausanne, Switzerland, under a specific protocol TIL-ME study with the number 247/13. The subsequent samples were collected by using the Pre-IT protocol (2016-02094).

Informed consent was obtained from any patients undergoing surgery at the CHUV. Patients were approached and requested to consent to donating their samples for translational research if the samples were not required for clinical pathological evaluation. There is no tissue selection based on patient sex, gender, history, age, previous treatments and thus no potential selection bias exists. The population characteristics were blinded to researchers.

For the melanoma cohort, we re-analysed results already published from a phase 1 trial of ACT with TILs in patients with melanoma (ClinicalTrials.gov ID NCT03475134)^11,12^. For correlation of PGE_2_ in the supernatant and TIL expansion, we collected supernatant of TIL cultures from patients enrolled in a phase I trial of ACT with TILs in solid tumours (CHUV-DO-0018-NeoTIL-2019; ClinicalTrials.gov ID NCT04643574).

The reported work was carried out in conformity with the Helsinki Declaration, and the protocol was authorized by the ethics committee of the canton of Vaud (Switzerland). Prior to the collection of study materials, all patients provided written informed consent. Tumour samples were processed and stored as previously described^47^.

### Mouse experiments

All mice were housed in a conventional animal facility of University of Lausanne and kept in individually ventilated cages, between 19–23 °C with 45–65% humidity and a 12 h dark/light cycle. All studies were approved by the Veterinary Authority of the Canton of Vaud and performed in accordance with Swiss ethical guidelines. No statistical methods were used to predetermine sample size. Sample sizes for in vivo assays were determined empirically based on previous work. For Winn assay, mice were randomly allocated to the different treatment groups based on weight of the mice while for the mouse adoptive cell therapy tumour control experiment, mice were randomized based on tumour size. Mouse experiments were performed blind to experimental conditions.

### Tumour processing and TIL expansion

For conventional pre-REP TIL expansion, freshly received solid tumour specimens were minced into 1 2 mm^2^ fragments and plated in a 48-well plate in 500 μl RPMI + 10% FBS + 1% PS containing 6,000 IU ml^−1^ IL-2 (Proleukin)^47^. Medium was refreshed twice a week and TILs splitted when confluent. In some cases, PGE_2_ at 1 μM, 10 μM EP2 (TG4-155, Cayman) and EP4 (ONO-AE3–208, ONO Pharmaceuticals) inhibitors, 11.7 μM ketorolac (TORA-DOL, 30 mg ml^−1^) were added once to the cultures at day 0. TG4-155 is a potent, brain-permeant and selective EP2 receptor antagonist with an inhibition constant (*K*_i_) of 9.9 nM and 550- to 4,750-fold selectivity for EP2 over EP1, EP3 and EP4^48^. ONO-AE3-208 is an orally active EP4-selective antagonist (*K*_i_ of 1.3 nM for EP4, 30 nM for EP3 and more than 10 μM for the prostanoid receptors EP1 and EP2)^49^.

Non-adherent plates were used to avoid fibroblast overgrowth in response to PGE_2_, which would affect TIL expansion. After 14–28 days of pre-REP TIL expansion, expanded TILs were counted, used for in vitro assays, or further expanded in REP phase for 14 days by using 40 Gy irradiated peripheral blood mononuclear cells (PBMCs) as feeder cells, 30 ng ml^−1^ OKT3 and 3,000 IU ml^−1^ IL-2.

### Blood processing and generation of RA T cells

PBMCs from leukapheresis of healthy donors were isolated by Ficoll gradient. Peripheral blood lymphocytes (PBLs) were enriched from PBMC using a pan T cell isolation kit (Miltenyi, 130-096-535) according to the manufacturer’s instructions. To generate RA T cells, PBLs were stimulated with CD3/CD28 beads (Dynabeads, 11132D) at 1:1 ratio for 10 days, changing beads every 3–4 days for a total of 3 stimulations in low-dose 60 IU ml−1 IL-2^50^. ‘Unstimulated’ T cells were cultured two days with 60 IU ml^−1^ IL-2.

### Protocol for the generation of mouse T cells overexpressing PGC1α and mouse RA T cells

OT-1-CD45.1 mice were obtained from and maintained in a conventional animal facility at the University of Lausanne following institutional guidelines. This study was approved by the Veterinary Authority of the Canton of Vaud (under license 6387) and performed in accordance with Swiss ethical guidelines. All mice (female) were housed in a conventional animal facility of University of Lausanne and kept in individually ventilated cages, between 19–23 °C with 45–65% humidity and a 12 h dark/light cycle. For mouse T cell PGC1α overexpression experiment, CD8^+^ OT-1 T cells were isolated from the spleens of OT-1 mice using CD8^+^ T cell Isolated Kit (MojoSortTM, 480044). CD8^+^ T cells (1 × 10^6^ cells per ml) were seeded into 24-well plates with a volume of 2 ml per well. Unstimulated CD8^+^ T cells were activated by treatment with anti-CD3 (2 μg ml^−1^, Invitrogen, 16-0031-86), anti-CD28 (1 μg ml^−1^, Invitrogen, 16-0281-86), and IL-2 (10 ng ml^−1^, Pepro Tech, AF-200-02-1000) concurrently for 72 h. Cells were then transduced with scramble or PGC1α-overexpressing retroviral construct^51^. Cells were stimulated as per protocol.

CD8^+^ OT-1 T cells were purified from the spleen of OT-1-CD45.1 mouse using a negative selection using the EasySep Mouse T cell Isolation kit (Stemcell Ref 19851).

Purified OT-1 CD8^+^ T cells were cultured in complete T cell medium: RPMI 1640 Glutamax supplemented with 10% heat-inactivated FBS, 100 UI ml^−1^ penicillin, 100 μg ml^−1^ streptomycin, 1 mM Hepes, 10 mM non-essential amino acids and 50 μM β-mercaptoethanol. Unstimulated, OT-1 T cells were cultured in T cell medium containing human IL-7/IL-15 at 5 ng ml^−1^. The medium was refreshed every 2 days. For repeatedly stimulation, OT-1 T cells were first stimulated with anti-CD3/CD28 beads (2 beads per cell) in the presence of 5 UI ml^−1^ of IL-2 during the first 3 days of culture and with IL-7/IL-15 at 5 ng ml^−1^ for 2 extra days. At day 5 after culture initiation, anti-CD3/CD28 beads were removed, and cells were re-stimulated at a concentration of 1 × 10^6^ cells per ml in the presence of 10 ng ml^−1^ SIINFEKL OVA peptide. OVA peptide stimulation was repeated on day 6 and 7. At day 8, unstimulated cells and RA T cells were collected for downstream experiments.

### Winn-type assay

NOD SCID common gamma KO mice were obtained from and maintained in a conventional animal facility at the University of Lausanne following institutional guidelines and kept in individually ventilated cages between 19–23 degrees with 45–65% humidity and a 12 h dark/light cycle. This study was approved by the Veterinary Authority of the Canton of Vaud (under license 3623b) and performed in accordance with Swiss ethical guidelines. All animals (male mice) were used at ages of 11 weeks. Control or COXi-expanded TILs were mixed with fresh autologous melanoma tumour cells in a 1:1 (T cell:tumour) ratio. A total of 1 × 10^6^ total cells (5 × 10^5^ tumour cells+ 5 × 10^5^ TILs) were subcutaneously injected into the right flank of each mouse (11-week-old) in 100 µl of PBS. Mice were monitored three times per week, and tumour volumes were calculated using the formula: *V*  =  (*L* × *W*^2^)/2. Mice were euthanized once tumours reached 1,000 mm^3^, or, according to regulation, if they became distressed, moribund or the tumour became necrotic. As negative controls, cancer cells (5 × 10^5^ cells) were injected with PBS.

### NSG in vivo tumour homing

IL-2 NOG mice (Taconic Biosciences) were maintained in a conventional animal facility at the University of Lausanne following institutional guideline and kept in individually ventilated cages, between 19–23 °C with 45–65% humidity and a 12 h dark/light cycle. This study was approved by the Veterinary Authority of the Canton of Vaud (under license 3746) and performed in accordance with Swiss ethical guidelines. Six- to nine-week-old female mice were anaesthetized with isoflurane and subcutaneously injected with 1 × 10^6^ human melanoma cells. At day 14, autologous 9 × 10^6^ control and COXi TILs were injected in the retro-orbital vein. At day 14 post-ACT, mice were euthanized by CO_2_ inhalation. Tumours were collected and dissociated in RPMI 1640 GlutaMAX with 0.3 PZ activity units per ml of collagenase and 30 IU ml^−1^ of pulmozyme (Roche) for 1 h at 37 °C, 5% CO_2_ on an orbital shaker. After digestion, tumour specimen suspension was filtered through a 100-µM nylon cell strainer and washed with PBS. Dissociated cells were then labelled for flow cytometry analysis.

### Cell culture and in vitro assays

Cells were maintained at 37 °C in complete medium R-10: RPMI 1640 supplemented with 2 mM l-glutamine, and 100 μg ml^−1^ penicillin and 100 U ml^−1^ streptomycin and 10% (v/v) heat-inactivated FBS or 8% human serum for T cells and TILs respectively. All T cell cultures were performed under low-dose 60 IU ml^−1^ IL-2 if not otherwise specified. For in vitro treatment of cells, MHY1485 (100 nM, Sigma-Aldrich, SML0810), everolimus (50 nM, Sigma-Aldrich, SML2282), 10 mM NAC, 100 μM vitamin E, 50 μM BAPTA-AM or 100 μM Rp-8-CPT cAMP analogue were added to the cultures. For metabolic experiments and metabolomics, Human Plasma-Like Medium (HPLM, A4899101, Gibco) was used. Cell count was performed using the AccuChip Kit with the ADAM-MC (NanoEntek) automated counter using propidium iodide to identify healthy cells per the manufacturer instruction.

Autologous tumour cell lines for tumour recognition assay were established from primary tumours by the Center of Experimental Therapies at CHUV. All cell lines were tested and negative for Mycoplasma.

### Cell viability assay

Fifty thousand TILs were seeded on 96-well plates and treated with PGE_2_ (1 µM). MCC950 (pyroptosis), Fst1 (ferroptosis), zVAD-FMK (apoptosis) and necrostatin 1S (necroptosis) inhibitors were added along with PGE_2_ at the indicated doses. Cell viability was assessed 72 h after the treatment using Cell Counting Kit 8 (WST-8/CCK8) (ab228554) as an indicator of viable cells. The cell viability was expressed as relative values compared to the control sample, which was defined as 100%. MCC950 (5381200001) was purchased from Sigma-Aldrich; Fst1 (17729) was purchased from Cayman; zVAD-FMK (ALX-260-02) was purchased from Enzo Life Sciences; and Nec 1 s (2263) was purchased from BioVisiony.

### In vitro tumour recognition assay

Expanded REP TILs were rested for 2 days in 60 IU ml^−1^ IL-2. Autologous tumour cell lines were plated at 70–90% confluence in a flat-bottom 96-well plate and let to adhere overnight. One million TILs were added to the wells for overnight co-culture. TILs were then collected, and tumour recognition was assessed via flow cytometry analysis to quantify 41BB surface expression (CD137) or cytokine secretion by TNF and IFNγ intracellular staining. Cells were cultured with 1× Brefeldin A (eBiosciences, 00-4506-51) for cytokine secretion measurement.

### RNA isolation and quantitative real-time PCR

Total RNA was isolated from 1 × 10^6^ cells with Trizol reagent (Life Technologies), followed by RNA purification using the RNA Easy Mini Kit (Qiagen). After treatment with RNase-free DNase I, 1 μg of total RNA was reverse-transcribed using PrimeScript First Strand cDNA Synthesis Kit (Takara Bio) as indicated by manufacturer. Quantitative real-time PCR was performed using TaqMan Fast Universal PCR reagents according to the manufacturer’s instructions. PCR amplification of the housekeeping gene *GAPDH* was performed for each sample as a control to allow normalization among samples. Each sample was run in triplicate, and each PCR experiment included three non-template control wells. The following primers were used: *IL2RA* (Hs00158122_m1), *IL2RB* (Hs01081697_m1), *IL2RG* (Hs00415671_m1), *PTGER2* (Hs00168754_m1), *PTGER3* (Hs00168755_m1), *PTGER4* (Hs00168761_m1), *PGC1A* (Hs00173304_m1), *PGC1B* (Hs00993805_m1), *HIF2A* (Hs01026149_m1), *CREB3L3* (Hs00962115_m1), *CPT1A* (Hs00912671_m1), *GPX4* (Hs00989766_g1), *Myb* (Hs00920556_m1), *TCF7* (Hs01556515_m1), *GAPDH* (Hs02786624_g1), *ACSL4 (Hs00244871), LPCAT3 (Hs01553683), FSP1* (Hs00210845) and *GLS2* (Hs00998733).

### RNA sequencing and data analysis

RNA from unstimulated and RA CD8^+^ T cells was extracted using the RNA easy kit, and RNA quality was assessed using a Fragment Analyzer. RNA-sequencing libraries were prepared for Illumina TruSeq Stranded Total RNA reagents according to the manufacturer’s instruction. Cluster generation was performed with the libraries using the Illumina HiSeq PE Cluster Kit v4 cBot reagents and sequenced on the Illumina HiSeq 4000 SR using HiSeq SBS Kit V4 reagents. The Illumina Pipeline Software version 1.84 was used to process the sequencing data.

Illumina paired-end sequencing reads were aligned to the human reference GRCh37.75 genome using STAR aligner (version 2.6.0c) and the two-pass method as briefly follows: the reads were aligned in a first round using the --runMode alignReads parameter, then a sample-specific splice-junction index was created using the --runMode genomeGenerate parameter. Finally, the reads were aligned using this newly created index as a reference. The number of counts was summarized at the gene level using htseq-count (version 0.9.1). The Ensembl ID were converted into gene symbols using the biomaRt package (version 2.58.1) and only protein-coding, immunoglobulin and TCR genes were conserved for the analysis. Read counts were normalized into reads per kilobase per million (RPKM) and log_2_-transformed after addition of a pseudo-count value of 1. Differential expression analyses were performed using the limma (v3.54.0). The PGE_2_ signature was derived from the top 63 significant (FDR-corrected) upregulated genes in CD8^+^ PBLs upon PGE_2_ treatment. Pathways scores were generated using ssGSEA from the R-package GSVA (version 1.44.5).

### scRNA-seq and scTCR-seq in TIL-ACT patients

Thirteen patients were enrolled in a phase I trial designed to test the feasibility of ACT with TILs (ClinicalTrials.gov ID NCT03475134). Two datasets profiling the TME by scRNA-seq (13 patients sorted for viable cells) and matched scRNA-seq and scTCR-seq data (13 patients sorted for CD45^+^ cells) were used as described^11^. We computed gene signature scores in CD8^+^ T cells for reactome pathways taken from MSigDB (https://www.gsea-msigdb.org/gsea/msigdb/; extracted from the C2 collection) by using the AUCell R package. The IL-2 signalling signature was extracted from Reactome under the ‘REACTOME_INTERLEUKIN_2_SIGNALING’ name. The PGE_2_ signature score was computed using the AUCell function and using as gene signature the 63 genes significantly upregulated by PGE_2_ treatment (versus control in RA CD8^+^ T cells, adjusted *P* value < 0.05, FC > 1). The PGE_2_ signature score was then computed for each cell. In patient-level analyses, signature scores were averaged pseudobulked per patient. CD8^+^ T cells were classified in several categorized according to their tumour-reactivity and ‘expanded in ACT product’ status. These two categories were defined as follows: annotated CD8^+^ clonotypes with their validated tumour-reactivity and their expansion profiles (using bulk TCRβ sequencing of the ACT product). Both categories, clinical responses and related data are fully described in Chiffelle et al.^12^.

### TCR cloning and tumour reactivity validation

Tumour reactivity interrogation from expanding TILs of the ACT products of the patients with melanoma and methodology was previously described^12^. In brief, TCRαβ pairs were cloned into recipient activated T cells or Jurkat cell line (TCR/CD3 Jurkat-luc cells (NFAT), Promega, stably transduced with human CD8αβ and TCRαβ CRISPR-KO). Full-length codon-optimized DNA sequences including TCR mouse constant regions were synthesized at GeneArt (Thermo Fisher Scientific) or Telesis Bio. DNA served as template for in vitro transcription and polyadenylation of RNA molecules as per the manufacturer’s instructions (Thermo Fisher Scientific). Autologous T cells were activated with Dynabeads Human T Activator CD3/CD28 beads (Thermo Fisher Scientific) at a ratio of 0.75 beads: 1 total PBMC in the presence of 50 IU mL^−1^ IL-2 (Proleukin). After 3 days of incubation at 37 °C and 5% CO_2_, beads were removed and activated T cells rested for 2 days before use. To transfect TCRαβ pairs into T cells and Jurkat cells, the Neon electroporation system (Thermo Fisher Scientific) was used, following the manufacturer’s instructions. In brief, cells were mixed with 300 ng of TCRα chain RNA together with 300 ng of TCRβ chain RNA and electroporated with the following parameters: 1,600 V, 10 ms, 3 pulses and 1,325 V, 10 ms, 3 pulses, for T and Jurkat cells, respectively. To assess antitumour-reactivity, 10^5^ TCR RNA-electroporated cells and 2 × 10^4^ to 10^5^ autologous tumour cells pre-treated with IFNγ were co-cultured in 96-wells plate. After overnight incubation, T cells were recovered and the upregulation of CD137 in T cells was evaluated by staining with anti-CD137 (Miltenyi), anti-CD3 (Biolegend or BD Biosciences), anti-CD4 (BD Biosciences), anti-CD8 (BD Biosciences) and anti-mouse TCRβ-constant (Thermo Fisher Scientific) and with Aqua viability dye (Thermo Fisher Scientific). With Jurkat cells, the luciferase assay was performed using the Bio-Glo Luciferase Assay System (Promega). The LSRFortessa (BD Bioscience) and IntelliCyt iQue Screener PLUS (Bucher Biotec) flow cytometers were used for acquisition. Fluorescence-activated cell sorting (FACS) data analysis was performed with FlowJo v10 (TreeStar). Luminescense was measured with a Spark Multimode Microplate Reader (Tecan).

### Bulk TCRβ sequencing and TCR repertoire analysis

Bulk TCRβ sequencing of the ACT products from control and COXi-expanded TILs was performed as previously described^52^. In brief, TILs mRNA was isolated and amplified using commercially available kits (from Life Technologies and Ambion, respectively) with the following modifications: in vitro transcription was performed at 37 °C for 16 h. First-strand cDNA was synthesized using the Superscript III (Thermo Fisher) and a collection of TRAV- or TRBV-specific primers. TCRs were then amplified by PCR with a single primer pair binding to the constant region and the adapter linked to the TRAV or TRBV primers added during the reverse transcription. A second round of PCR was performed to add the Illumina adapters containing the different indexes. The TCR products were purified, quantified and loaded on the MiniSeq instrument (Illumina) for deep sequencing of the TCRβ chain. The TCR sequences were further processed using ad hoc Perl scripts to: (1) pool all TCR sequences coding for the same protein sequence; (2) filter out all out-of-frame sequences; (3) determine the abundance of each distinct TCR sequence. TCRs with a single read were not considered for the analysis. Richness was assessed by the number of unique TCR sequences present in the repertoire. The clonality was described by the Shannon Entropy or 1-Pielou’s evenness^7^. 10x and 100x expanded (Exp) clones refers to the number of clonotypes with a frequency 10 or 100-fold higher than the median frequency of the repertoire.

### Western blot

T cell pellet was lysed with RIPA Lysis and Extraction Buffer (Thermo Fisher) supplemented with protease and phosphatase inhibitors. Protein concentrations were quantified using a Quick Start Bradford assay kit (BioRad). Samples containing 20 μg of protein in NuPAGE LDS Sample Buffer (4×) were separated using 8–12% pre-cast SDS–PAGE gels (BioRad). Proteins were transferred to PVDF membranes and blocked for 1 h in 5% Milk in TBST buffer. PVDF membranes were incubated overnight at 4 °C in 1% BSA TBST buffer with the following primary antibodies at dilution 1:1,000 or otherwise specified: β-actin (K2713, Santa Cruz, sc-47778, 1:2000), JAK1 (B-3 Santa Cruz sc-376996, 1:500), pJAK1 (D7N4Z Cell Signaling 74129), JAK3 (B-12, Santa Cruz, sc-6932, 1:500), pJAK3 (D44E3, Cell Signaling, 5031), STAT1 (D4Y6Z, Cell Signaling, 14995), pSTAT1 (D4A7, Cell Signaling, 7649), STAT3 (D3Z2G, Cell Signaling, 12640), pSTAT3 (D3A7, Cell Signaling, 9145), STAT5 (D206Y Cell Signaling 94205), pSTAT5 (D47E7, Cell Signaling, 9351), AKT (C67E7, Cell Signaling, 4691), pAKT (D9E, Cell Signaling, 4060), mTOR (7C10, Cell Signaling, 2983), pmTOR (D9C2, Cell Signaling, 5536), S6 (5G10, Cell Signaling, 2217), pS6 (D57.2.2E, Cell Signaling, 4858), PGC1a (3G6 Cell Signaling, 2178 s), GPX4 (EPNC1R144, Abcam, 125066).

PVDF membranes were washed 3 times for 10 min at room temperature in TBST buffer and incubated with appropriate secondary horseradish peroxidase-linked antibodies for 1 h at room temperature with 1:10,000 dilution in 1% BSA in TBST buffer: anti-mouse HRP (Dako, p0447), anti-goat HRP (Dako, P0449), anti-rabbit HRP (Dako, P0448). Blots were developed on Fusion FX imaging system (Vilber) using ECL plus reagent (GE Healthcare). Densitometry analysis was performed in ImageJ. Images of all uncut blots can be found in Supplementary Figs. 2 and 3.

### Flow cytometry

The following antibodies were used at dilution 1:50 for staining cells for flow cytometry: CD45 (BV570, HI30 Biolegend, 304034), CD4 (BV605, OKT4, Biolegend, 317438), CD8 (BV650, RPA-T8, Biolegend, 301042), Tim3 (APC fire 750, F38-2E2, Biolegend, 345044), CTLA4 (PE, BNI3, Biolegend, 369604), PD-1 (BV421, EH12.2H7, Biolegend, 329920), CD39 (BV711, TU66, BD Bioscience, 563680), Lag3 (AF488, 11C3C65, Biolegend, 369326), Ki67 (PE–Cy7, Ki67, Biolegend, 350526), Ki67 (AF700, B56, BD Bioscience, 561277), CD57 (BV605, QA17A04, Biolegend, 393304), TOX/TOX2 (PE, E6G50, Cell Signaling, 25202), TOX (PE, REA473, Miltenyi, 130-120-716), CD28 (AF700, CD28.2, Biolegend, 302920), CD27 (APC–Cy7, M-T271, Biolegend, 356424), IL-2Rα (FITC, BC96, Biolegend, 302604), CD122 (PE, TU27, Biolegend, 339006), CD132 (APC, TUGh4, Biolegend, 338608), TCF1/TCF7 (AF647, C63D9, Cell Signaling, 6932), CD56 (PE–Cy7, 5.1H11, Biolegend, 362510), CD137 (PE-Cy5, 4B4-1, Biolegend 309808), CD3 (BV510, UCHT1, Biolegend, 300448), CD3 (BV711, UCHT1, BD Bioscience, 563725), CD4 (PE-CF594, RPA-T4, BD Bioscience, 562281), pS6 (PE, cupk43k, eBioscience, 12-9007-42), IFNγ (APC, B27, Biolegend, 506510), TNF (PE–Cy7, MAb11, BD Bioscience, 557647).

For flow cytometry phenotyping analysis, T cells were washed with phosphate-buffered saline (PBS) and stained with Zombie UV fixable (Biologend, Cat:423107) or LIVE/DEAD Fixable Aqua Dead Cell Stain Kit (Thermo Fisher, L34957) for 15 min on ice in PBS. Cells were subsequently stained for surface markers in PBS + 2% FBS for 15 min on ice. For intracellular staining, eBioscience Foxp3/Transcription Factor kit was used (Thermo Fisher Scientific, 00-5523-00). Cells were fixed 1 h in fix/perm buffer (Thermo Fisher) and intracellular staining was performed for 45 min at room temperature in perm buffer. After staining, cells were acquired on a four-laser Fortessa (BD Biosciences) with FACS DIVA software v.9.0 (BD Biosciences) and analysed with FlowJo (TreeStar).

For mitochondrial mass and membrane potential staining, cells were incubated for 20 min at 37 °C in an incubator with 25 nM TMRM (Thermo Fisher Scientific, M20036) and 100 nM Mitotracker Green (Thermofisher, M46750) in medium before staining, respectively.

Lipid peroxidation was assessed via Bodipy 581/591 C11 (Thermofisher, D3861). Cells were incubated for 30 min at 37 °C with 2 μM Bodipy 581/591 C11.

To assess proliferation, cells were stained for 7 min in 1 μM CFSE and subsequently washed with PBS + 2% FBS to quench the reaction.

The MitoSOX Mitochondrial Superoxide Indicator (MitoSOX) was used to quantify mitochondria superoxide production following manufacturer’s instructions.

O-propargyl-puromycin (OPP) in TILs was measured with Click-iT Plus OPP Alexa Fluor 488 Protein Synthesis Assay Kit (Thermo Fisher, C10456).

For the analysis of the effect of PGE_2_ on downstream signalling pathways (IL-2, CD3, CD3/CD28), pre-REP TILs were cultured for 48 h in the absence of IL-2. On the day of the experiment, TILs were washed and resuspended for 2 h in RPMI with or without PGE_2_ (1μM). TILs were then washed and stimulated during 30 min with IL-2 (100 IU ml^−1^) alone, coated anti-CD3 (0.5 μg ml^−1^) alone, or combinations of anti-CD3/anti-CD28 in RPMI medium complemented with the following labelling antibodies: CD3 (BioLegend), CD8 (BioLegend) and Aqua viability dye (Thermo Fisher). TILs were then fixed with paraformaldehyde (PFA, 1.6%) for 10 min at room temperature, washed and permeabilized with methanol on ice for 30 min (99.9%). TILs were then washed in PBS 1% BSA and labelled with phosphorylated ribosomal protein S6 (pS6, eBioscience). After staining, cells were acquired on a three-laser iQue Screener PLUS (Sartorius) with iQue ForeCyt software v.6.2 (Sartorius) and analysed with FlowJo X (TreeStar).

For profiling of tumour antigen-specific COXi-treated versus control TILs, cells were labelled with an in-house MART-1 (A27L) HLA*A0201-multimer (ELAGIGILTV, developed by the Peptide and Tetramer Core Facility of the Department of Oncology, UNIL-CHUV, Lausanne, Switzerland).

Representative flow cytometry gating strategies for analysis of CD4^+^ or CD8^+^ PBLs or TILs can be found in Supplementary Fig. 1a,b.

### FACS sorting of TILs for metabolic flux reconstruction

Tumours were dissociated as described^11^, and single-cell suspension samples were stained for FACS sorting. Viable single cells TILs were gated using RedDot-1 /DAPI, CD4 and CD8 markers as depicted in Supplementary Fig. 1c and used for further analyses.

### Mass cytometry acquisition and analysis

For the profiling of COXi-treated versus control products, REP TILs were stained with 34 metal-labelled antibodies (Standard BioTools and in house, described in Supplementary Table 5). Cells were first incubated with a 5 μM solution of cisplatin in PBS for viability assessment. Cells were then washed and resuspended in Maxpar Cell Staining Buffer (MCSB, Standard BioTools) with human Fc-receptor blocking solution (Miltenyi) (10 min at room temperature). Following surface staining (30 min at room temperature), cells were then fixed using Cytofix fixation buffer (12 min at room temperature) (BD Biosciences) and permeabilized using Phosflow Perm Buffer III solution (20 min at 4 °C) (BD Biosciences). Intracellular staining was then performed (30 min at room temperature). Cells were next incubated with cell intercalation solution (Standard BioTools) (overnight at 4 °C).

Cells were then washed, resuspended in Maxpar Cell Acquisition Solution MCAS (Standard BioTools) containing EQ Four Element Calibration Beads and filtered into cell strainer cap tubes, immediately prior to CyTOF data acquisition. Data were acquired on a Helios Mass Cytometer (Standard BioTools). Raw mass cytometry data were normalized with the bead passport EQ from the CyTOF Software version 7 (Standard BioTools). Data were then pre-processed using FlowJo v10 (TreeStar) and selected live cells were exported for further analysis.

The following antibodies were used for staining cells for CyTOF at specified dilutions: Granzyme B (106 Cd, GB11, Abcam ab103159, 1:100), Ki67 (111 Cd, B56, Abcam, ab279657, 1:100), granzyme (K145Nd, GM6C3, Santa cruz, sc-56125, 1:200), TCF1 (150Nd, 7F11A10, Biolegend, 655202, 1:100), Eomes (154Sm, WD1928, Invitrogen, 14-4877-82, 1:100), p-p38 (156Gd, D3F9, Standart BioTools, 3156002 A, 1:50), TOX (159Tb, REA, Miltenyi, 130-126-455, 1:100), Tbet (161Dy, 4B10, Standart BioTools, 3161014B, 1:200), FoxP3 (162Dy, PCH101, Standart BioTools, 3162011 A, 1:50), KLRG1 (166Er, SA231A2, Biolegend, 367702, 1:100), CTLA4 (170Er, 14D3, Standart BioTools, 3170005B, 1:50), CD45 (089Y, HI30, Standart BioTools, 3089003B, 1:400), CD57 (110 Cd, HCD57, Standart BioTools, MBS140192, 1:100), CD8a (112 Cd, RPA-T8, Biolegend, 301053, 1:100),CD4 (113 Cd, RPA-T4, Biolegend, 300502, 1:100), HLA-DR (114 Cd, L243, Biolegend, 307602, 1:100), CD3 (141Pr, UCHT1, Standart BioTools, 3141019B, 1:100), OX40 (142^Nd^, ACT35, Standart BioTools, 3142018B, 1:50), CD45RA (143Nd, HI100, Standart BioTools, 3143006B, 1:200), CCR5 (144Nd, NP-6G4, Standart BioTools, 3144007 A, 1:200), CD28 (146Nd, CD28.2, Biolegend, 302937, 1:100), CD127 (149Sm, A019D5, Standart BioTools, 3149011B, 1:200), CD103 (151Eu, Ber-ACT8, Standart BioTools, 3151011B, 1:100), TIM-3 (153Eu, F38-2E2, Standart BioTools, 3153008B, 1:200), IL-2Rα (155Gd, 2A3, Biolegend, 356102, 1:100), CD27 (158Gd, L128, Standart BioTools, 3158010B, 1:400), CD39 (160Gd, A1, Standart BioTools, 3160004B, 1:100), CXCR3 (164Dy, G025H7, Biolegend, 353702, 1:100), CCR7 (167Er, G043H7, Standart BioTools, 3167009 A, 1:100), ICOS (169Tm, C398.4 A, Standart BioTools, 3169030B, 1:200), 41BB (173Yb, 4B4-1, Standart BioTools, 3173015B, 1:200), PD-1 (174Yb, EH12.2H7, Standart BioTools, 3174020B, 1:100), LAG3 (175Lu, 11C3C65, Standart BioTools, 3175033B, 1:100), CD56 (176Yb, NCAM16.2, Standart BioTools, 3176008B, 1:400), Viability (Cis-pt,Standard BioTools, 201064) and DNA (195-Ir, Standart BioTools 201192 A).

Subsequently, a hierarchical gating strategy was implemented with the openCyto library, resulting in the generation of three distinct populations: the ‘root’ population, the CD4^hi^CD8^low^ population, and the CD4^low^CD8^hi^ population, labelled as CD8^+^ TILs in cumulative figures. For clustering within the CD4^low^CD8^hi^ population, FlowSOM was applied to ensure balanced clustering and each sample was sub-sampled within these populations to a maximum of 150,000 cells. The data underwent clustering using FlowSOM and ConsensusClusterPlus, resulting in metaclusters. The optimal number of metaclusters (11 clusters) was determined based on the average Silhouette width.

Based on the metaclustering results, separation between the COXi-treated and control TILs was visualized the using UMAP. Additionally, violin plots were then created and that belonging to specific clusters (1, 2, 3, 8 and 9) for both COXi-treated and control conditions were used. Cohen’s *D* effect sizes were calculated for each comparison to assess the significance of differences.

### Polychromatic imaging cytometry

For Imagestream analysis, live TILs at 1 × 10^7^ cells per ml were run at 100 cells per second on the ImageStreamX MarkII (Merck Millipore). TILs were stained with relevant antibodies for CD4, CD8, IL-2Rα, IL-2Rβ, IL-2Rγ_c_ and DAPI. Single stained cells were used as compensation controls. Images were captured at 60× magnification. Data were analysed using the ImageStream Data Analysis and Exploration Software (IDEAS). Colocalization was calculated based on Bright Detail Similarity score, a log-transformed Pearson’s correlation coefficient computed by Amnis.

### Confocal microscopy and dSTORM

Cells were plated in chamber slides at a 70%–80% confluence. Following PGE_2_ treatment, cells were washed with PBS and fixed with PFA for 8 min at room temperature. After blocking with 5% BSA, fixed cells were incubated overnight at 4 °C with primary antibodies. Secondary antibodies were incubated for 1 h at room temperature. Nuclei were counterstained with DAPI (2 μg ml^−1^ in PBS) for 5 min at room temperature. Slides were then mounted using Fluoromount-G.

Colocalization was quantified following this analysis procedure: Circular patches surrounding a cell or a group of cells or manual evaluation of IL-2Rβγ_c_ levels (region of interest) and non- IL-2Rβγ_c_ signal (random region of interest) were selected. Colocalization values were calculated using a pixel-wise Pearson’s test. Frequency quantification of Pearson’s test values (−1: opposing, 0: no and 1: maximum colocalization).

Immunofluorescence staining was performed on formalin-fixed paraffin-embedded (FFPE) tumoural tissue sections. Four µm-thick FFPE sections were subjected to routine deparaffinization and rehydration, blocked with 5% BSA and incubated overnight at 4 °C with primary antibodies. Secondary antibodies were incubated for 1 h at room temperature. Nuclei were counterstained with DAPI (2 μg ml^−1^ in PBS) for 5 min at room temperature. Slides were then mounted using Fluoromount-G.

LipidSpot (Biotium, 70065-T) was used following manufacturing protocol on live cells for lipid droplet imaging. Cells were mounted in Vectashield HardSet Mounting Medium.

Proximity ligation assay was performed using Duolink PLA (Sigma) for IL-2Rβ and IL-2Rγ_c_ using manufacturer’s instruction. Manual evaluation of IL-2Rβ–γ_c_ signal or circular patches surrounding a cell or a group of cells were draw to assess the numbers of dots from the IL-2Rβ–γ_c_ signal. All samples were imaged on a Zeiss LSM 780 confocal microscope and analysed using ImageJ.β

For dSTORM, 25 mm round coverslips (Marienfeld, 1.5H, 0117650) were washed with ethanol plasma cleaned for 30 s and coated with poly-l-lysine (Sigma, P8920). Cells wμere seeded on coverslips, fixed with 4% PFA, and stained according to standard immunofluorescence staining with primary antibodies against IL2Rγ and IL2Rβ and secondary antibodies goat anti-mouse AF647 and goat anti-rabbit AF555 (Thermofisher A-21241 and A-21428). Coverslips were mounted using an 35 mm adapter (Okolabs, RA-35-18-2000-06) and covered with 450 μl of STORM buffer (Idylle, KMO-ETE-450-IDY, Everspark 1.0). 555 and 642 lasers were aligned before each round of imaging. Imaging was performed on a Zeiss Elyra 7 microscope with the 63× oil-immersion Plan APOCHROMAT objective (Zeiss, NA 1.46, 1.6× lens). Alexa647 and Alexa555 dyes were imaged in sequential time-series of approximately 20,000 frames each. Both molecules were ground-state depleted and in ultra-high power mode.For each dye, ground-state return was elicited by continuous illumination with a 405 laser. Images were recorded with an Andor iXon + 897 EMCCD. 2D dSTORM data analysis and visualization were carried out with the Zen Black 3.0 SR software (Zeiss) as previously described^53^.

### Fluorescence resonance energy transfer

FRET assays included IL-2Rγ_c_–Bv510 as a donor and IL-2Rβ–PE as an acceptor. FACS-FRET measurements were performed using a FACSLSRII SORP (BD Bioscience) equipped with 355 nm, 405 nm, 488 nm, 561 nm and 633 nm lasers. To measure IL-2Rγ_c_ (donor) signal and FRET, cells were excited with the 405 nm laser and fluorescence was collected in the BV510 channel with a standard 530/30 filter, while the FRET-signal was measured with a 586/15 filter. To measure IL-2Rβ (acceptor) signal, cells were excited with the 561 nm laser while the emission was also taken with a 586/15 filter. For each sample, we evaluated a minimum of 250 positive cells that fell within the background-adjusted gate.

### Ca^2+^ signalling

TILs were loaded with 1 μM Fluo-4 (Thermo Fisher) for 30 min at 37 °C, cells were then washed and resuspended in loading medium (RPMI + 10% FCS) in a poly-l-lysine-coated coverslips mounted in a RC-20 closed bath chamber (Warner Instrument). Fluorescence was excited at 490 nm and detected at >515 nm, with an acquisition rate of 10 Hz. The Fluo-4-loaded cells were treated or not with PGE_2_ (1 μM), after which healthy cells were identified by their responsiveness to 1 μM ionomycin (Calbiochem). Single-cell video images were obtained on a Nikon Ti2 spinning-disk microscope.

### Determination of mitochondrial DNA copy number

Mitochondrial DNA Copy number of CD8^+^ T cells and control or COXi-expanded TILs was determined as previously mentioned^54^. Quantitative real-time PCR (rtPCR) was performed using KAPA SYBR FAST qPCR Kit Master Mix on a QuantStudio 6 Flex Real-Time PCR System (Thermo Fisher) after total DNA was extracted using Genomic-tip 20/G (QIAGEN) (KAPA Biosystems).

### ELISA

PGE_2_ level in supernatant from expanded TILs was determined using a human PGE_2_ ELISA Kit (abcam, ab287802) according to the manufacturer’s instructions. For the solid tumour cohort, PGE_2_ levels were measured by ELISA during the first medium change. For the breast and melanoma cohort PGE_2_ levels were measured between day 7 and 10. Total and reduced glutathione levels were determined with the GSH + GSSG/GSH Assay Kit (Colorimetric) (abcam, ab239709). Intracellular ATP levels were quantified with ATP Detection Assay Kit (ab113849). Intracellular MDA levels were measured with the TBARS fluorometric microplate assay (FR45, Oxford Biomedical Research).

### Electron microscopy

Unstimulated and RA T cells were fixed in glutaraldehyde solution (EMS) 2.5% in phosphate buffer (PB 0.1 M (pH 7.4)) for 1 h at room temperature and post-fixed in a fresh mixture of osmium tetroxide 1% (EMS) with 1.5% of potassium ferrocyanide (Sigma) in PB buffer for 1 h at room temperature. The samples were washed twice in distilled water and dehydrated in ethanol solution (Sigma, St Louis) at graded concentrations (30%, 40 min; 50%, 40 min; 70%, 40 min; 100%, 2 × 1 h). This was followed by infiltration in Spurr resin (EMS) at graded concentrations (Spurr 33% in ethanol, 4 h; Spurr 66% in ethanol, 4 h; Spurr 100%, 2 × 8 h) and finally polymerized for 48 h at 60 °C in an oven. Ultrathin sections of 50 nm thickness were cut transversally at 2, 5 and 6 mm from the root tip and at 2 mm below the hypocotyl-root junction, using a Leica Ultracut (Leica Mikrosysteme), picked up on a copper slot grid 2 × 1 mm (EMS) coated with a polystyrene film (Sigma). Sections were post-stained with uranyl acetate (Sigma) 4% in H_2_O for 10 min, rinsed several times with H_2_O, followed by Reynolds lead citrate in H_2_O (Sigma) for 10 min and rinsed several times with H_2_O. Micrographs were taken with a transmission electron microscope Philips CM100 (Thermo Fisher Scientific) at an acceleration voltage of 80 kV with a TVIPS TemCamF416 digital camera (TVIPS) using the software EM-MENU 4.0 (TVIPS). Panoramic alignments were performed with the software IMOD.

### Seahorse XFe96 metabolic flux analysis

Oxygen consumption rate was measured at 37 °C using an XFe96 extracellular analyser (Seahorse Bioscience). Twenty-four hours before the experiments, PGE_2_ (1 μM) was added to the cultures. On the day of the assay, T cells were plated in Seahorse XFe96 Microplates (2 × 10^5^ cells per well) previously coated with Cell-Tak (22.4 mg ml^−1^), using Seahorse medium supplemented with glucose (10 mM), pyruvate (1 mM) and glutamine (2 mM). Mitochondrial function was interrogated by the sequential injection of oligomycin (1.5 μM, ATP synthetase inhibitor), FCCP (0.5 μM, uncoupling agent) and antimycin A (0.5 μM, complex III inhibitor) in combination with rotenone (0.5 μM, complex I inhibitor), following standard Seahorse XFe96 protocol. Every point represents an average of *n* = 6 per experiment.

### Reconstruction of a metabolic model for T cells

We generated a reduced model around the metabolic subsystems of interest to study the effect of PGE_2_ in CD8^+^ RA T cells and TILs. To this end, we applied the redHUMAN method25 to the human genome-scale metabolic network Recon 3D^55^. We used the composition of the RPMI medium to define the extracellular medium in the model, and we allowed all the inorganic metabolites to be uptaken or secreted.

We selected 45 starting subsystems, namely aminosugar metabolism, arachidonic acid metabolism, arginine and proline metabolism, cholesterol metabolism, chondroitin sulfate degradation, chondroitin synthesis, citric acid cycle, coA catabolism, coA synthesis, eicosanoid metabolism*, fatty acid oxidation*, fatty acid synthesis*, fructose and mannose metabolism, galactose metabolism, glutamate metabolism, glutathione metabolism, glycerophospholipid metabolism, glycine, serine, alanine, and threonine metabolism, glycolysis/gluconeogenesis, glycosphingolipid metabolism, haem synthesis, heparan sulfate degradation, inositol phosphate metabolism, keratan sulfate degradation, keratan sulfate synthesis, leukotriene metabolism, linoleate metabolism, methionine and cysteine metabolism, miscellaneous*, NAD metabolism, nucleotide interconversion*, pentose phosphate pathway, phenylalanine metabolism, purine synthesis, pyrimidine synthesis, pyruvate metabolism, ROS detoxification, sphingolipid metabolism, starch and sucrose metabolism, steroid metabolism, tetrahydrobiopterin metabolism, triacylglycerol synthesis, urea cycle, oxidative phosphorylation, and all the mitochondrial reactions. For subsystems tagged with an asterisk, we included only the part that was deregulated by PGE_2_. We used the redHUMAN parameters, *D* = 1 for redGEM, Smin for redGEMX, and Sminp3 for lumpGEM. As a result, we reconstructed redTcellPGE2, a metabolic model with 2,602 metabolites, 1,898 genes and 5,051 reactions associated with 81 metabolic subsystems.

### Transcriptomics data integration to generate a metabolic context-specific CD8^+^ T cell model

Based on the experimental data, we assumed a maximum doubling time of 24 h for the RA T cells and TILs and 36 h when cultured with PGE_2_.

We identified in the RNA-seq data 1,072 metabolic genes present in the redTcellPGE2 model. We computed the averaged fold change for each condition (RA PGE_2_ versus RA control) for the three replicates. We evaluated the gene–protein–reaction rules in the metabolic model to assign the gene expression to the corresponding enzymes. Next, we classified the enzymes into up- or downregulated using a threshold of 1.3. Therefore, enzymes with a fold change above 1.3 are considered upregulated, and enzymes with a fold change below 0.77 are considered downregulated. The 1,072 genes code for 2,722 reactions, of which 491 are upregulated, and 55 are downregulated based on the transcriptomics data.

In order to integrate the data into the redTcellPGE2 model, we used the method REMI^56^, which integrates transcriptomics data into metabolic models, assuming that deregulations in the gene expression translate to deregulations of the corresponding enzyme abundance and, therefore, in the reaction rate. In particular, REMI imposes constraints so that if the gene associated with a reaction is upregulated, its reaction rate must be higher. Conversely, if the gene is downregulated, the reaction rate will be lower. Then the method maximizes the number of reaction rates simultaneously constrained in the network according to the fold change of the corresponding gene expression between the two conditions^12^.

From the REMI results, we observed that out of the 546 deregulated reactions, a maximum of 425 reaction rates could be simultaneously constrained in the network according to their expression profile. We identified 19 alternative sets of 425 reactions and 399 reactions that are common across alternatives.

### Metabolic reaction fluxes representative of each CD8^+^ RA T cell treatment

We fixed the ratios of the 399 reactions consistent with the network and used the Artificial Centering Hit-and-Run sampler (ACHR) to sample 100,000 points from the solution space. We then computed for each reaction the mean rate and the mean fold change of the populations of samples between both conditions, and we used them as representative for the analysis of the metabolic state of RA T cells with PGE_2_.

### Metabolic reaction fluxes representative of each TIL treatment

Similar to the reconstruction of RA T cell specific models treated with PGE_2_, we reconstructed TIL-specific models by integrating scRNA-seq data for TILs into the redTcellPGE2 model (Supplementary Table 3). We first identified 60 deregulated metabolic genes in TILs treated or not with PGE_2_ (selecting genes with a threshold of at least 1.2-fold change and *P* values up to 0.5). Next, we used REMI to integrate the data into the metabolic redTcellPGE2 model. Subsequently, we mapped the 60 deregulated genes to 111 metabolic reactions and we identified with the computational analyses that 86 of these fluxes could be simultaneously constrained in the network according to the corresponding deregulation profile. We then sampled 100,000 points from the flux solution space and we computed the fold changes of each reaction rate for TILs treated or not with PGE_2_.

### Minimal network enrichment analysis to study metabolic functions

To study the effects of PGE_2_ on T cells’ metabolic functions, we defined seven metabolic tasks associated with the proliferation, energy, and production of ROS and lipid droplets. Using the composition of the biomass reaction in the model, we extracted the corresponding reactions representing the synthesis of the macromolecules, ATP and superoxide anion production, and the Recon 3D lipid droplet production reaction. To generate minimal networks for the metabolic tasks^27^, we formulated a mixed integer linear programme and identified the minimum number of reactions required to synthesize each metabolic task and possible alternatives.

Next, after integrating the transcriptomics data into the redTcellPGE2 model, we performed minimal network enrichment analysis (MiNEA) using either the gene expression data or the representative of the metabolic fluxes computed by sampling for RA T cells treated or not with PGE_2_.

### Metabolomics

Cell lysate from 5 different RA CD8^+^ T cells and sorted CD8^+^ TILs (*n* = 4) treated or not with 1 μM PGE_2_ for 24 h were pre-extracted and homogenized by adding 400 µl of methanol:H_2_O (4:1), in the Cryolys Precellys 24 sample Homogenizer (2 × 20 s at 10,000 rpm, Bertin Technologies) with ceramic beads. The bead beater was air-cooled down at a flow rate of 110 l min^−1^ at 6 bar. Homogenized extracts were centrifuged for 15 min at 4,000*g* at 4 °C (Hermle). The resulting supernatant was collected and evaporated to dryness in a vacuum concentrator (LabConco). Dried sample extracts were resuspended in methanol:H_2_O (4:1, v/v) according to the total protein content.

The protein pellets were evaporated and lysed in 20 mM Tris-HCl (pH 7.5), 4 M guanidine hydrochloride, 150 mM NaCl, 1 mM Na2EDTA, 1 mM EGTA, 1% Triton, 2.5 mM sodium pyrophosphate, 1 mM β-glycerophosphate, 1 mM Na_3_VO_4_, 1 µg ml^−1^ leupeptin using the Cryolys Precellys 24 sample Homogenizer (2 × 20 s at 10,000 rpm, Bertin Technologies) with ceramic beads. BCA Protein Assay Kit (Thermo Scientific) was used to measure (*A*_562 nm_) total protein concentration (Hidex).

#### FFA analysis

Extracted samples were analysed by reversed phase liquid chromatography coupled to high-resolution mass spectrometry (RPLC-HRMS) operating in negative mode using a 6550 Ion-Funnel Q-TOF instrument interfaced with 1290 UHPLC system (Agilent Technologies). Chromatographic separation was carried out on a Zorbax Eclipse Plus C18 (1.8 μm, 100 mm × 2.1 mm internal diameter column) (Agilent Technologies). The mobile phase comprised 60:40 (v/v) acetonitrile:water with 10 mM ammonium acetate and 0.1% acetic acid (A) and 88:10:2 isopropanol: acetonitrile:water with 10 mM ammonium acetate and 0.1% acetic acid (B). The linear gradient elution from 15% to 30% B was applied for 2 min, then from 30% to 48% B for 0.5 min, from 48% to 72% B, and the last gradient step from 72% to 99% B followed by 0.5 min isocratic conditions and a 3 min re-equilibration to the initial chromatographic conditions. The flow rate was 600 μl min^−1^, with a column temperature of 60 °C and a sample injection volume of 2 µl. Electrospray ionization source conditions were set as follows: dry gas temperature 200 °C, nebulizer 35 psi and flow 14 l min^−1^, sheath gas temperature 300 °C and flow 11 l min^−1^, nozzle voltage 1,000 V, and capillary voltage −3,500 V. Full scan acquisition mode in the mass range of 100–1200 m/z was applied for data acquisition.

### Statistical analyses and reproducibility

All statistical tests were performed using R (version 3.3.0) and GraphPad Prism software (v8 and v9.3.1; GraphPad). Data points represent biological replicates and are shown as mean ± s.d. Statistical tests to derive *P* values were performed as specified in the figure legends. Metabolomics statistical analyses were performed using MetaboAnalyst v5.0^57^.

Data were collected using biological replicates to ensure reproducibility. The number of independent replicates for each experiment is noted in all figure legends.

### Reporting summary

Further information on research design is available in the Nature Portfolio Reporting Summary linked to this article.

## Online content

Any methods, additional references, Nature Portfolio reporting summaries, source data, extended data, supplementary information, acknowledgements, peer review information; details of author contributions and competing interests; and statements of data and code availability are available at 10.1038/s41586-024-07352-w.

### Supplementary information

Reporting Summary

Supplementary Fig. 1 Flow cytometry gating strategies. a,b, Flow cytometry gating strategy for analysis of single viable CD4^+^CD8^+^ PBLs (a) or single viable CD4^+^CD8^+^ TILs (b). c, Gating strategy applied for sorting of CD4^+^/CD8^+^ TILs from dissociated tumours for metabolic flux reconstruction study.

Supplementary Fig. 2 Western blots for IL-2 signalling. Uncut western Blots from IL-2 signalling in RA T cells treated with PGE_2_ for 48h and subsequently stimulated with IL-2 or an IL-2Rβγ_c_ (IL-2v) mutein for 15 min. β-actin controls are depicted on the right.

Supplementary Fig. 3 Western blots for PGC1a and GPX4 expression. Uncut western blots from GPX4 protein expression in TILs treated or not with PGE_2_ or Fst1 (top) and PGC1-α protein expression upon IL-2 stimulation in presence/absence of PGE_2_ (bottom). β-actin controls are depicted on the right.

Supplementary Table 1 PGE_2_ signature. Top 63 significant (FDR-corrected) upregulated genes in CD8^+^ PBLs upon PGE_2_ treatment.

Supplementary Table 2 Bulk RNA-seq unstimulated and RA CD8^+^ T cells. Fold change gene expression in unstimulated and RA CD8^+^ T cells upon PGE_2_ treatment.

Supplementary Table 3 Flux reconstruction in TILs. Fold change gene expression upon PGE_2_ treatment in CD8^+^ TILs used for metabolic flux reconstruction analysis.

Supplementary Table 4 CD8^+^ TIL metabolomics. Metabolite quantification in CD8^+^ TILs upon PGE_2_ treatment.

Supplementary Table 5 CyTOF markers. List of 34 markers used for mass cytometry analysis.

Supplementary Table 6 Tumour growth WINN assay. Tumour growth kinetic in control or COXi TIL-treated mice.

## Data Availability

Transcriptomic data generated in this study have been deposited in Gene Expression Omnibus (GEO) under accession number GSE227316. Differential gene expression analyses derived from bulk RNA-seq analysis of PBLs, pseudobulked data from scRNA-seq analysis of TILs and metabolomics data are provided as Supplementary Tables 2–4.
