# Supplementary material for: PGE2 inhibits TIL expansion by disrupting IL-2 signalling and mitochondrial function
Source: Nature. 2024 Apr 24;629(8011):426–34. doi: 10.1038/s41586-024-07352-w (PMC11078736; doi:10.1038/s41586-024-07352-w)
Supplement: Supplementary file 1 — Reporting Summary [file 41586_2024_7352_MOESM1_ESM.pdf]

nature portfolio

Corresponding author(s): George Coukos and Denarda Dangaj Laniti

Last updated by author(s): Mar 13, 2024

Reporting Summary

Nature Portfolio wishes to improve the reproducibility of the work that we publish. This form provides structure for consistency and transparency in reporting. For further information on Nature Portfolio policies, see our Editorial Policies and the Editorial Policy Checklist.

Statistics

For all statistical analyses, confirm that the following items are present in the figure legend, table legend, main text, or Methods section.

n/a	Confirmed	
☐	☒	The exact sample size (*n*) for each experimental group/condition, given as a discrete number and unit of measurement
☐	☒	A statement on whether measurements were taken from distinct samples or whether the same sample was measured repeatedly
☐	☒	The statistical test(s) used AND whether they are one- or two-sided<br>*Only common tests should be described solely by name; describe more complex techniques in the Methods section.*
☒	☐	A description of all covariates tested
☐	☒	A description of any assumptions or corrections, such as tests of normality and adjustment for multiple comparisons
☐	☒	A full description of the statistical parameters including central tendency (e.g. means) or other basic estimates (e.g. regression coefficient) AND variation (e.g. standard deviation) or associated estimates of uncertainty (e.g. confidence intervals)
☐	☒	For null hypothesis testing, the test statistic (e.g. *F*, *t*, *r*) with confidence intervals, effect sizes, degrees of freedom and *P* value noted<br>*Give P values as exact values whenever suitable.*
☒	☐	For Bayesian analysis, information on the choice of priors and Markov chain Monte Carlo settings
☒	☐	For hierarchical and complex designs, identification of the appropriate level for tests and full reporting of outcomes
☐	☒	Estimates of effect sizes (e.g. Cohen's *d*, Pearson's *r*), indicating how they were calculated

Our web collection on statistics for biologists contains articles on many of the points above.

Software and code

Policy information about availability of computer code

Data collection	The following softwares were used for data collection:<br>ADAM-MC Automated Cell counter (NanoEntek)<br>Illumina HiSeq 4000 SR (Illumina)<br>iQue ForeCyt software v.6.2 (Sartorius)<br>Spark Multimode Microplate Reader (Tecan)<br>FACS DIVA software v.9.0 (BDbiosciences)<br>CyTOF Software version 7 (Standard BioTools)<br>EM-MENU 4.0 (TVIPS GmbH, Gauting, Germany)<br>XFe96 extracellular analyser (Seahorse Bioscience)<br>Fusion FX imaging system (Vilber)<br>Zen software (Zeiss)<br>ImageStream Data Analysis and Exploration Software (IDEAS, Merck Millipore).<br>QuantStudio 6 Flex Real-Time PCR System (ThermoFisher)<br>1290 UHPLC system (Agilent Technologies)
Data analysis	The following softwares were used to data analysis:<br>Graph design and statistical analysis were performed using GraphPad Prism v8 and v9.3.1<br>Flow cytometry analysis data were analysed by FACS DIVA software v.9.0 (BDbiosciences) and FlowJo software v.10.4 (BDbiosciences)<br>Imaging flow cytometry data were analysed usind IDEAS software V.6 (Millipore)<br>MetaboAnalyst (version 5.0)<br>MATLAB Mathworks (R2021b)

CPLEX IBM (v12.10)
ImageStream Data Analysis and Exploration Software (IDEAS, Merck Millipore)
Gene Set Enrichment Analysis (GSEA, version 4.3.2)
Softwares used for Mass Cytometry data analysis: FlowJo (version 10.4), openCyto_2.14.0 library (https://doi.org/10.1371/journal.pcbi.1003806), FlowSOM_2.1.0 (https://doi.org/10.1002/cyto.a.22625), ConsensusClusterPlus_1.66.0(https://doi.org/10.1093/bioinformatics/btq170), umap_0.2.10.0 (https://doi.org/10.21105/joss.00861).
Illumina Pipeline Software version 1.84 (Illumina)
R version 3.3.0 with packages: STAR aligner (version 2.6.0c) , htseq-count (version 0.9.1), biomaRt (version 2.58.1) , edgeR R (version 3.38.4), GSVA (version 1.44.5), AUCell (1.18), limma (v3.54.0)
Wave Controller 2.4.3 (Seahorse Bioscience)
Zen software v.3.7. (Zeiss)
Zen Black 3.0 SR software (Zeiss)
ImageJ V1.54h
EM-MENU 4.0 (TVIPS GmbH, Gauting, Germany)

For manuscripts utilizing custom algorithms or software that are central to the research but not yet described in published literature, software must be made available to editors and reviewers. We strongly encourage code deposition in a community repository (e.g. GitHub). See the Nature Portfolio guidelines for submitting code & software for further information.

Data

Policy information about availability of data

All manuscripts must include a data availability statement. This statement should provide the following information, where applicable:

- Accession codes, unique identifiers, or web links for publicly available datasets
- A description of any restrictions on data availability
- For clinical datasets or third party data, please ensure that the statement adheres to our policy

Transcriptomic data generated in this study have been deposited in Gene Expression Omnibus (GEO) under accession numbers GSE227316 (https://www.ncbi.nlm.nih.gov/geo/query/acc.cgi?acc=GSE227316).
Differential gene expression analysis derived from bulkRNAseq analysis of PBLs, pseudobulked data from scRNAseq analysis of TILs and metabolomics data are provided as supplementary tables S2, S4 and S5.

Datasets:
Reactome and Hallmarks collections were extracted from MSigDB (https://www.gsea-msigdb.org/gsea/msigdb/; extracted from the C2 collection)
Tumour-reactivity interrogation from expanding TILs of the ACT products of the melanoma: Chiffelle et al. (https://www.biorxiv.org/content/10.1101/2023.07.21.544585v1) and the bulk TCR data from the ACT product are available in the Gene Expression Omnibus (GEO) under the GSE234352 accession number.
Profiling the TME of melanoma patients (n=13) by scRNA-seq and matched scRNA-seq/scTCR-seq were used as described in Barras et al. (DOI: 10.1126/sciimmunol.adg7995) and the data are available in GEO under the GSE222448 accession number.

Access to custom code:
No new custom code was generated. For scRNA-seq, TCR-seq data analysis data were analysed as in Barras et al. (DOI: 10.1126/sciimmunol.adg7995). Model reconstruction was performed as described in Methods and in Masid et al. (doi:10.1038/s41467-020-16549-2) and metabolic flux analysis was performed as described in Pandey et al.(doi:10.1371/journal.pcbi.1006760, doi:10.1371/journal.pcbi.1007036).

Human research participants

Policy information about studies involving human research participants and Sex and Gender in Research.

Reporting on sex and gender	Sex, gender and age were not considered in the study design and this information was not collected as part of our protocol
Population characteristics	Tumour samples were collected from individuals with :<br>melanoma: 38<br>non–small cell lung cancer: 6<br>ovarian cancer: 9<br>breast cancer: 33
Recruitment	Tumour samples were collected between October 2016 and August 2023 at the Centre Hospitalier Universitaire Vaudoise (CHUV), Lausanne, Switzerland was under a specific protocol TIL-ME study with the number 247/13. After that, samples were collected by using the Pre-IT protocol (2016-02094).<br>Informed consent was obtained from any patients undergoing surgery at the CHUV. Patients were approached and requested to consent to donating their samples for translational research if the samples were not required for clinical pathological evaluation. There is no tissue selection based on patient history, age, previous treatments and thus no potential selection bias exists. The population characteristics were blinded to researchers.<br>For the analysis of the melanoma cohort, we re-analyzed results already published from a phase 1 trial of ACT with TILs in melanoma patients (ClinicalTrials.gov NCT03475134). For correlation of PGE2 in the supernatant and TIL expansion, we used collected supernatant of TIL cultures from patients enrolled in a phase 1 trial of ACT with TILs in solid tumours (CHUV-DO-0018-NeoTIL-2019, NCT04643574).
Ethics oversight	The reported work was carried out in conformity with the Helsinki Declaration, and the protocol was authorised by the ethics committee of the canton of Vaud (Switzerland). Prior to the collection of study materials, all patients provided written informed consent.

Note that full information on the approval of the study protocol must also be provided in the manuscript.

Field-specific reporting

Please select the one below that is the best fit for your research. If you are not sure, read the appropriate sections before making your selection.

☒ Life sciences ☐ Behavioural & social sciences ☐ Ecological, evolutionary & environmental sciences

For a reference copy of the document with all sections, see nature.com/documents/nr-reporting-summary-flat.pdf

Life sciences study design

All studies must disclose on these points even when the disclosure is negative.

Sample size	No statistical methods were used to predetermine sample size. Sample sizes for in vitro and in vivo assays were determined empirically based on previous work and minimum of 3 biological replicates were used in most of the studies to allow for statistical comparisons.
Data exclusions	No data were excluded from our analysis
Replication	Data was collected using biological replicates to ensure reproducibility. The number of independent replicates for each experiment is noted in all figure legends. All experimental findings were reproduced succesfully at least 2 times.
Randomization	For in vitro experiments, no randomization was performed. For murine Winn assay, mice were randomly allocated to the different treatment groups based on weight of the mice while for the murine adoptive cell therapy tumor control experiment, mice were randomized based on tumor size.
Blinding	Blinding was not conducted for most in vitro research because the same people performed the experiments, collected the data, and analysed it. Imaging flow cytometry analysis, Cytoff analysis and electron microscopy acquistion and analysis were performed blindly.<br>Murine experiments were performed blind to experimental conditions.

Reporting for specific materials, systems and methods

We require information from authors about some types of materials, experimental systems and methods used in many studies. Here, indicate whether each material, system or method listed is relevant to your study. If you are not sure if a list item applies to your research, read the appropriate section before selecting a response.

Materials & experimental systems

n/a	Involved in the study	
☐	☒	Antibodies
☐	☒	Eukaryotic cell lines
☒	☐	Palaeontology and archaeology
☐	☒	Animals and other organisms
☐	☒	Clinical data
☒	☐	Dual use research of concern

Methods

n/a	Involved in the study	
☒	☐	ChIP-seq
☐	☒	Flow cytometry
☒	☐	MRI-based neuroimaging

Antibodies

Antibodies used

Flowcytometry:
Zombie UV fixable Viability kit BUV395 Part: 77474 Biolegend 423108
LIVE/DEAD™ Fixable Aqua Dead Cell Stain Kit ThermoFisher L34957
CD45 BV570 HI30 Biolegend 304034, 1:50
CD4 BV605 OKT4 Biolegend 317438, 1:50
CD8 BV650 RPA-T8 Biolegend 301042, 1:50
Tim3 APC fire 750 F38-2E2 Biolegend 345044, 1:50
CTLA4 PE BNI3 Biolegend 369604, 1:50
PD1 BV421 EH12.2H7 Biolegend 329920, 1:50
CD39 BV711 TU66 BD horizon 563680, 1:50
Lag3 AF488 11C3C65 Biolegend 369326, 1:50
Ki67 PECy7 Ki-67 Biolegend 350526, 1:50
TOX/TOX2 PE E6G50 Cell Signaling 25202, 1:50
TOX PE REA473 Miltenyi 130-120-716, 1:50
CD28 AF700 CD28.2 Biolegend 302920, 1:50
CD27 APC Cy7 M-T271 Biolegend 356424, 1:50
CD25 FITC BC96 Biolegend 302604, 1:50

CD122 PE TU27 Biolegend 339006, 1:50
CD132 APC TUGh4 Biolegend 338608, 1:50
TCF1 / TCF7 AF647 C63D9 Cell Signaling 6932, 1:50
CD56 pe cy7 5.1H11 Biolegend 362510, 1:50
CD137 pe cy5 4B4-1 Biolegend 309808, 1:50
CD3 BV510 UCHT1 Biolegend 300448, 1:50
CD3 BV711 UCHT1 BD 563725, 1:50
CD4 PE-CF594 RPA-T4 BD 562281, 1:50
CD57 BV605 QA17A04 Biolegend 393304, 1:50
Ki67 AF700 B56 BD 561277, 1:50
IFNg APC B27 Biolegend 506510, 1:50
TNFa PECy7 MAb11 BD Bioscience 557647, 1:50
pS6 PE cupk43k eBioscience 12-9007-42, 1:50
Reddot-1 Far red 40060 Biotium, 1:200

CyTOF:
Granzyme B 106Cd GB11 Abcam ab103159, 1:100
Ki-67 111Cd B56 Abcam ab279657, 1:100
granzyme K145Nd GM6C3 Santa cruz sc-56125, 1:200
TCF1 150Nd 7F11A10 Biolegend 655202, 1:100
Eomes 154Sm WD1928 Invitrogen 14-4877-82, 1:100
p-p38 156Gd D3F9 Standart BioTools 3156002A, 1:50
TOX 159Tb REA Miltenyi 130-126-455, 1:100
Tbet 161Dy 4B10 Standart BioTools 3161014B, 1:200
FoxP3 162Dy PCH101 Standart BioTools 3162011A, 1:50
KLRG1 166Er SA231A2 Biolegend 367702, 1:100
CTLA-4 170Er 14D3 Standart BioTools 3170005B, 1:50
CD45 089Y HI30 Standart BioTools 3089003B, 1:400
CD57 110Cd HCD57 Standart BioTools MBS140192, 1:100
CD8a 112Cd RPA-T8 Biolegend 301053, 1:100
CD4 113Cd RPA-T4 Biolegend 300502, 1:100
HLA-DR 114Cd L243 Biolegend 307602, 1:100
CD3 141Pr UCHT1 Standart BioTools 3141019B, 1:100
OX40 142Nd ACT35 Standart BioTools 3142018B, 1:50
CD45RA 143Nd HI100 Standart BioTools 3143006B, 1:200
CCR5 144Nd NP-6G4 Standart BioTools 3144007A, 1:200
CD28 146Nd CD28.2 Biolegend 302937, 1:100
CD127 149Sm A019D5 Standart BioTools 3149011B, 1:200
CD103 151Eu Ber-ACT8 Standart BioTools 3151011B, 1:100
TIM-3 153Eu F38-2E2 Standart BioTools 3153008B, 1:200
CD25 155Gd 2A3 Biolegend 356102, 1:100
CD27 158Gd L128 Standart BioTools 3158010B, 1:400
CD39 160Gd A1 Standart BioTools 3160004B, 1:100
CXCR3 164Dy G025H7 Biolegend 353702, 1:100
CCR7 167Er G043H7 Standart BioTools 3167009A, 1:100
ICOS 169Tm C398.4A Standart BioTools 3169030B, 1:200
4-1BB 173Yb 4B4-1 Standart BioTools 3173015B, 1:200
PD-1 174Yb EH12.2H7 Standart BioTools 3174020B, 1:100
LAG-3 175Lu 11C3C65 Standart BioTools 3175033B, 1:100
CD56 176Yb NCAM16.2 Standart BioTools 3176008B, 1:400
Viability Cis-pt Standart BioTools 201064
DNA 195-Ir Standart BioTools 201192A

Western blot:
B actin K2713 Santa Cruz sc-47778, 1:2000
JAK1 B-3 Santa Cruz sc-376996, 1:500
pJAK1 D7N4Z Cell signaling 74129, 1:1000
JAK3 B-12 Santa Cruz sc-6932, 1:500
pJAK3 D44E3 Cell signaling 5031, 1:1000
STAT1 D4Y6Z Cell signaling 14995, 1:1000
pSTAT1 D4A7 Cell signaling 7649, 1:1000
STAT3 D3Z2G Cell signaling 12640, 1:1000
pSTAT3 D3A7 Cell signaling 9145, 1:1000
STAT5 D206Y Cell signaling 94205, 1:1000
pSTAT5 D47E7 Cell signaling 9351, 1:1000
AKT C67E7 Cell signaling 4691, 1:1000
pAKT D9E Cell signaling 4060, 1:1000
mTOR 7C10 Cell signaling 2983, 1:1000
pmTOR D9C2 Cell signaling 5536, 1:1000
S6 5G10 Cell signaling 2217, 1:1000, 1:1000
pS6 D57.2.2E Cell signaling 4858, 1:1000
PGC1a 3G6 2178s Cell signaling, 1:1000
GPX4 EPNCIR144 125066, Abcam, 1:1000
anti-mouse HRP: Dako, p0447
anti-goat HRP: Dako, P0449

anti-rabbit HRP:: Dako, P0448

Validation

All antibodies used in this study are commercially available and have been validated by the manufacturers. For flowcytometry and western blots, antibodies were titrated or used at concentration suggested by manufacturer.
Gates were set using unstained and isotype control antibodies and/or just secondary antibodies

Zombie UV fixable Viability kit BUV395 Part: 77474 Biolegend 423108
https://www.biolegend.com/fr-ch/products/zombie-uv-fixable-viability-kit-9336?GroupID=BLG2181

LIVE/DEAD™ Fixable Aqua Dead Cell Stain Kit ThermoFisher L34957
https://www.thermofisher.com/order/catalog/product/L34957?gclid=Cj0KCQiAgqGrBhDtARIsAM5s0_neD8cGTnIRUBdB7_CPtEPHvi9dKW9BnxgSeMBPkbGwNJbqYgA_b9oaAss0EALw_wcB&s_kwcid=AL!3652!3!606658601267!e!!g!!live%20dead%20fixable%20aqua&ef_id=Cj0KCQiAgqGrBhDtARIsAM5s0_neD8cGTnIRUBdB7_CPtEPHvi9dKW9BnxgSeMBPkbGwNJbqYgA_b9oaAss0EALw_wcB:G:s&s_kwcid=AL!3652!3!606658601267!e!!g!!live%20dead%20fixable%20aqua!381166034!75094237911&cid=bid_pca_frg_r01_co_cp1359_pjt0000_bid00000_0se_gaw_bt_pur_con&gad_source=1

CD45 BV570 HI30 Biolegend 304034
https://www.biolegend.com/nl-nl/products/brilliant-violet-570-anti-human-cd45-antibody-7409

CD4 BV605 OKT4 Biolegend 317438
https://www.biolegend.com/en-us/products/brilliant-violet-605-anti-human-cd4-antibody-7820?GroupID=BLG5901

CD8 BV650 RPA-T8 Biolegend 301042
https://www.biolegend.com/fr-lu/productstab/brilliant-violet-650-anti-human-cd8a-antibody-7652

Tim3 APC fire 750 F38-2E2 Biolegend 345044
https://www.biolegend.com/en-ie/search-results/apc-fire-750-anti-human-cd366-tim-3-antibody-13878

CTLA4 PE BNI3 Biolegend 369604
https://www.biolegend.com/nl-nl/products/pe-anti-human-cd152-ctla-4-antibody-12897

PD1 BV421 EH12.2H7 Biolegend 329920
https://www.biolegend.com/fr-ch/products/brilliant-violet-421-anti-human-cd279-pd-1-antibody-7191?GroupID=BLG5466

CD39 BV711 TU66 BD horizon 563680
https://www.bdbiosciences.com/en-us/products/reagents/flow-cytometry-reagents/research-reagents/single-color-antibodies-ruo/bv711-mouse-anti-human-cd39.563680

Lag3 AF488 11C3C65 Biolegend 369326
https://www.biolegend.com/en-us/products/alexa-fluor-488-anti-human-cd223-lag-3-antibody-15130

Ki67 PECy7 Ki-67 Biolegend 350526
https://www.biolegend.com/nl-nl/products/pe-cyanine7-anti-human-ki-67-antibody-9084

TOX/TOX2 PE E6G50 Cell Signaling 25202
https://www.cellsignal.com/products/antibody-conjugates/tox-tox2-e6g5o-rabbit-mab-pe-conjugate/25202

TOX PE REA473 Miltenyi 130-120-716
https://www.miltenyibiotec.com/CH-en/products/tox-antibody-anti-human-mouse-reafinity-rea473.html#conjugate=pe:size=100-tests-in-200-ul

CD28 AF700 CD28.2 Biolegend 302920
https://www.biolegend.com/en-us/search-results/alexa-fluor-700-anti-human-cd28-antibody-3435?GroupID=BLG5919

CD27 APC Cy7 M-T271 Biolegend 356424
https://www.biolegend.com/fr-ch/products/apc-cyanine7-anti-human-cd27-antibody-12841

CD25 FITC BC96 Biolegend 302604
https://www.biolegend.com/en-ie/products/fitc-anti-human-cd25-antibody-615

CD122 PE TU27 Biolegend 339006
https://www.biolegend.com/nl-nl/products/pe-anti-human-cd122-il-2rbeta-antibody-5624

CD132 APC TUGh4 Biolegend 338608
https://www.biolegend.com/nl-be/products/apc-anti-human-cd132-common-gamma-chain-antibody-5561

TCF1 / TCF7 AF647 C63D9 Cell Signaling 6932
https://www.cellsignal.com/products/antibody-conjugates/tcf1-tcf7-c63d9-rabbit-mab-alexa-fluor-647-conjugate/6709

CD56 pe cy7 5.1H11 Biolegend 362510
https://www.biolegend.com/en-us/products/pe-cyanine7-anti-human-cd56-ncam-antibody-9959?GroupID=BLG13037

CD137 pe cy5 4B4-1 Biolegend 309808
https://www.biolegend.com/en-us/products/pe-cyanine5-anti-human-cd137-4-1bb-antibody-3909?GroupID=BLG2203

CD3 BV510 UCHT1 Biolegend 300448
https://www.biolegend.com/ja-jp/products/brilliant-violet-510-anti-human-cd3-antibody-9792?GroupID=BLG5900

CD3 BV711 UCHT1 BD 563725
https://www.bdbiosciences.com/en-us/products/reagents/flow-cytometry-reagents/research-reagents/single-color-antibodies-ruo/bv711-mouse-anti-human-cd3.563725

CD4 PE-CF594 RPA-T4 BD 562281
https://www.bdbiosciences.com/en-nz/products/reagents/flow-cytometry-reagents/research-reagents/single-color-antibodies-ruo/pe-cf594-mouse-anti-human-cd4.562316

CD57 BV605 QA17A04 Biolegend 393304
https://www.biolegend.com/nl-be/search-results/brilliant-violet-605-anti-human-cd57-recombinant-antibody-15480

Ki67 AF700 B56 BD 561277
https://www.bdbiosciences.com/en-au/products/reagents/flow-cytometry-reagents/research-reagents/single-color-antibodies-ruo/alexa-fluor-700-mouse-anti-ki-67.561277

pS6 PE cupk43k eBioscience 12-9007-42
https://www.thermofisher.com/antibody/product/Phospho-S6-Ser235-Ser236-Antibody-clone-cupk43k-Monoclonal/12-9007-42

Reddot-1 Far red 40060 Biotium, 1:200
https://biotium.com/product/reddottm1-far-red-nuclear-stain-200x-in-h2o/

IFNg APC B27 Biolegend 506510, 1:50
https://www.biolegend.com/en-us/products/apc-anti-human-ifn-gamma-antibody-1533

TNFa PECy7 MAb11 BD Bioscience 557647, 1:50
https://www.bdbiosciences.com/en-us/products/reagents/flow-cytometry-reagents/research-reagents/single-color-antibodies-ruo/pe-cy-7-mouse-anti-human-tnf.557647

B actin K2713 Santa Cruz sc-47778
https://www.scbt.com/fr/p/beta-actin-antibody-c4

JAK1 B-3 Santa Cruz sc-376996
https://www.scbt.com/fr/p/jak1-antibody-b-3

pJAK1 D7N4Z Cell signaling 74129
https://www.cellsignal.com/products/primary-antibodies/phospho-jak1-tyr1034-1035-d7n4z-rabbit-mab/74129

JAK3 B-12 Santa Cruz sc-6932
https://www.scbt.com/fr/p/jak3-antibody-b-12

pJAK3 D44E3 Cell signaling 5031
https://www.cellsignal.com/products/primary-antibodies/phospho-jak3-tyr980-981-d44e3-rabbit-mab/5031

STAT1 D4Y6Z Cell signaling 14995
https://www.cellsignal.com/products/primary-antibodies/stat1-d4y6z-rabbit-mab/14995

pSTAT1 D4A7 Cell signaling 7649
https://www.cellsignal.com/products/primary-antibodies/phospho-stat1-tyr701-d4a7-rabbit-mab/7649

STAT3 D3Z2G Cell signaling 12640
https://www.cellsignal.com/products/primary-antibodies/stat3-d3z2g-rabbit-mab/12640

pSTAT3 D3A7 Cell signaling 9145
https://www.cellsignal.com/products/primary-antibodies/phospho-stat3-tyr705-d3a7-xp-rabbit-mab/9145

STAT5 D206Y Cell signaling 94205
https://www.cellsignal.com/products/primary-antibodies/stat5-d2o6y-rabbit-mab/94205

pSTAT5 D47E7 Cell signaling 9351
https://www.cellsignal.com/products/primary-antibodies/phospho-stat5-tyr694-antibody/9351

AKT C67E7 Cell signaling 4691
https://www.cellsignal.com/products/primary-antibodies/akt-pan-c67e7-rabbit-mab/4691

pAKT D9E Cell signaling 4060
https://www.cellsignal.com/products/primary-antibodies/phospho-akt-ser473-d9e-xp-rabbit-mab/4060

mTOR 7C10 Cell signaling 2983
https://www.cellsignal.com/products/primary-antibodies/mtor-7c10-rabbit-mab/2983

pmTOR D9C2 Cell signaling 5536
https://www.cellsignal.com/products/primary-antibodies/phospho-mtor-ser2448-d9c2-xp-rabbit-mab/5536

	S6 5G10 Cell signaling 2217<br>https://www.cellsignal.com/products/primary-antibodies/s6-ribosomal-protein-5g10-rabbit-mab/2217<br><br>pS6 D57.2.2E Cell signaling 4858<br>https://www.cellsignal.com/products/primary-antibodies/phospho-s6-ribosomal-protein-ser235-236-d57-2-2e-xp-rabbit-mab/4858<br><br>PGC1a 3G6 2178 Cell signaling<br>https://www.cellsignal.com/products/primary-antibodies/pgc-1a-3g6-rabbit-mab/2178<br><br>GPX4 EPNCIR144 125066<br>https://www.abcam.com/products/primary-antibodies/glutathione-peroxidase-4-antibody-epncir144-ab125066.html<br><br>All antibodies used for Mass Cytometry were validated and titrated to determine optimal concentrations.<br>Gates were set using unstained and MMO controls.

Eukaryotic cell lines

Policy information about cell lines and Sex and Gender in Research

Cell line source(s)	Autologous tumor cell lines for tumor recognition assay were established from primary tumors by the Center of Experimental Therapies at CHUV. No names were given to these cell lines. Sex from donor patients was not tracked in this study. No commercial available cell lines were used.
Authentication	No further authentification of the primary tumor lines has been performed for this study.
Mycoplasma contamination	All cell lines were tested and negative for Mycoplasma
Commonly misidentified lines<br>(See ICLAC register)	No commonly misidentified lines were used.

Animals and other research organisms

Policy information about studies involving animals; ARRIVE guidelines recommended for reporting animal research, and Sex and Gender in Research

Laboratory animals	All the mouse experiments were carried out with sex and age matched groups. All mice were housed in a conventional animal facility of University of Lausanne and kept in individually ventilated cages,between 19-23 degrees with 45-65% humidity and a 12hour dark/light cycle.<br><br>OT1 PGC1a overexpression: All animals (female mice) were used at ages of 6-7 weeks. The strains and source of mice: C57BL/6 OT1 CD45.1+mice were obtained from Pedro Romero's laboratory (UNIL).<br><br>NOD SCID common gamma KO (NOD.Cg-Prkdcscid Il2rgtm1Wjl/SzJ): All animals (male mice) were used at ages of 11 weeks. The strains and source of mice: NOD SCID common gamma KO mice were obtained from the UNIL animal facility (Epalinges).<br><br>hIL-2 NOG (NOD.Cg-Prkdcscid Il2rgtm1Sug Tg(CMV-IL2)4-2Jic/JicTac): All animals (female mice) were used at ages of 6-9 weeks. The strains and source of mice: hIL-2 NOG mice were obtained from Taconic Biosciences.
Wild animals	No wild animals were involved
Reporting on sex	Female or male mice were used to match gender of T cell donor for adoptive transfer
Field-collected samples	No samples were collected from the field
Ethics oversight	All experiments were conducted according to Swiss federal regulation and approved by the veterinary authority of Canton Vaud.

Note that full information on the approval of the study protocol must also be provided in the manuscript.

Clinical data

Policy information about clinical studies
All manuscripts should comply with the ICMJE guidelines for publication of clinical research and a completed CONSORT checklist must be included with all submissions.

Clinical trial registration	NCT03475134, NCT04643574
Study protocol	ClinicalTrials.gov: NCT03475134, NCT04643574

Data collection	Tumour samples were collected between October 2016 and August 2023 at the Centre Hospitalier Universitaire Vaudoise (CHUV), Lausanne, Switzerland was under a specific protocol TIL-ME study with the number 247/13. After that, samples were collected by using the Pre-IT protocol (2016-02094).<br>Informed consent was obtained from any patients undergoing surgery at the CHUV. Informed consent was obtained from any patients undergoing surgery at the CHUV. Patients were approached and requested to consent to donating their samples for translational research if the samples were not required for clinical pathological evaluation. There is no selection based on previous history, age, previous treatments and thus no potential selection bias exists. The population characteristics were blinded to researchers.<br>For the analysis of the melanoma cohort, we re-analyzed results already published from a phase 1 trial of ACT with TILs in melanoma patients (ClinicalTrials.gov NCT03475134). For correlation of PGE2 in the supernatant and TIL expansion, we used collected supernatant of TIL cultures from patients enrolled in a phase 1 trial of ACT with TILs in solid tumours (CHUV-DO-0018-NeoTIL-2019, NCT04643574).
Outcomes	This study did not evaluate any clinical outcomes. Clinical samples from prior phase I clinical trials were only utilised in this project for translational purposes and described in Barras et al. (DOI: 10.1126/sciimmunol.adg7995).

Flow Cytometry

Plots

Confirm that:

☒ The axis labels state the marker and fluorochrome used (e.g. CD4-FITC).

☒ The axis scales are clearly visible. Include numbers along axes only for bottom left plot of group (a 'group' is an analysis of identical markers).

☒ All plots are contour plots with outliers or pseudocolor plots.

☒ A numerical value for number of cells or percentage (with statistics) is provided.

Methodology

Sample preparation	Cells analyzed by flowcytometry were derived from human dissociated tumour, expanded TILs and peripheral blood lymphocytes isolated from PBMC or murine T cells extracted from the spleen of OTI mice or from the tumours of tumours-bearing mice. Methods for cell isolation and staining are described in the material and method section.
Instrument	Cells were analyzed on Fortessa flow cytometer (BD Biosciences) and on IntelliCyt iQue® Screener PLUS (Bucher Biotec)
Software	BD FACSDiva was used for data acquisition. Flowjo v10.5.3 was used for data analysis.<br>ForeCyt® was used for data acquisition on IntelliCyt iQue® Screener PLUS.
Cell population abundance	Cell population abundance was quantified based on the frequency of parents, or otherwise specified in the axis labels.
Gating strategy	CD8+ or CD4+ T cells were gated based on the following gating strategy: SSC-A/FSC-A, singlet (FSC-A/FSC-H), live cells (UV zombie negatif), CD45+, CD3+, CD8+/CD4+ as depicted in Supplementary figure 1.

☒ Tick this box to confirm that a figure exemplifying the gating strategy is provided in the Supplementary Information.
