# Supplementary material for: PGE2 inhibits TIL expansion by disrupting IL-2 signalling and mitochondrial function
Source: Nature. 2024 Apr 24;629(8011):426–34. doi: 10.1038/s41586-024-07352-w (PMC11078736; doi:10.1038/s41586-024-07352-w)
Supplement: Supplementary file 2 — Flow cytometry gating strategies. a,b, Flow cytometry gating strategy for analysis of single viable CD4+CD8+ PBLs (a) or single viable CD4+CD8+ TILs (b). c, Gating strategy applied for sorting of CD4+/CD8+ TILs from dissociated tumours for metabolic flux reconstruction study. [file 41586_2024_7352_MOESM2_ESM.pdf]

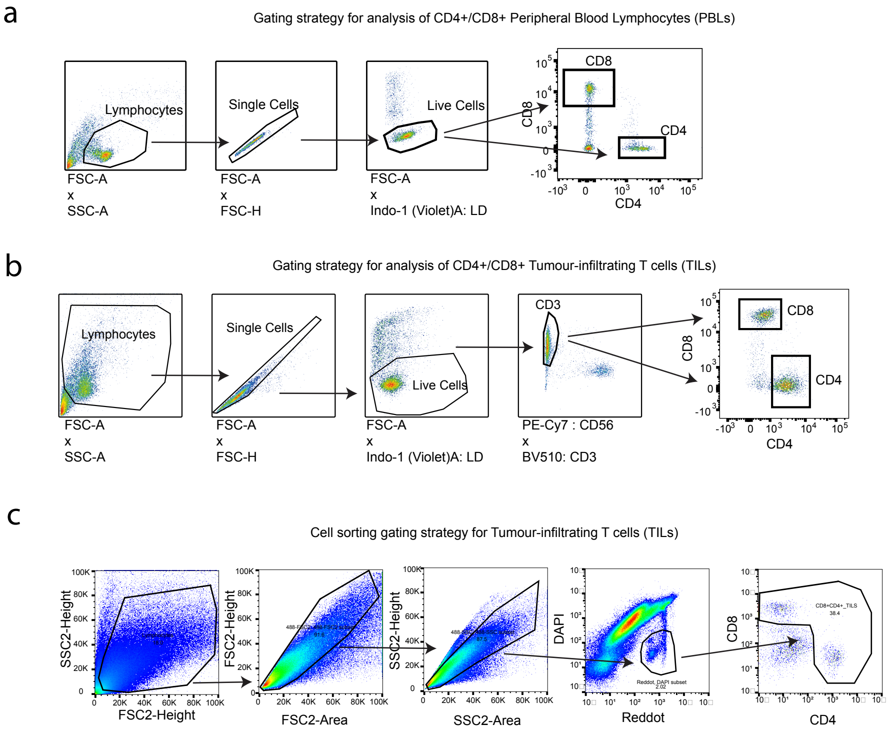
a
Gating strategy for analysis of CD4+/CD8+ Peripheral Blood Lymphocytes (PBLs)
Lymphocytes
FSC-A
x
SSC-A
Single Cells
FSC-A
x
FSC-H
Live Cells
FSC-A
x
Indo-1 (Violet)A: LD
CD8
CD4
b
Gating strategy for analysis of CD4+/CD8+ Tumour-infiltrating T cells (TILs)
Lymphocytes
FSC-A
x
SSC-A
Single Cells
FSC-A
x
FSC-H
Live Cells
FSC-A
x
Indo-1 (Violet)A: LD
CD3
PE-Cy7 : CD56
x
BV510: CD3
CD8
CD4
c
Cell sorting gating strategy for Tumour-infiltrating T cells (TILs)
SSC2-Height
FSC2-Height
FSC2-Height
FSC2-Area
SSC2-Height
SSC2-Area
DAPI
Reddot
Reddot, DAPI subset
2.02
CD8
CD4
CD8+CD4+_TILS
38.4
