# Supplementary material for: PGE2 inhibits TIL expansion by disrupting IL-2 signalling and mitochondrial function
Source: Nature. 2024 Apr 24;629(8011):426–34. doi: 10.1038/s41586-024-07352-w (PMC11078736; doi:10.1038/s41586-024-07352-w)

In Figure 2k

Boxes indicate regions shown in the figure.
β-actin band for each gel is shown on the right.

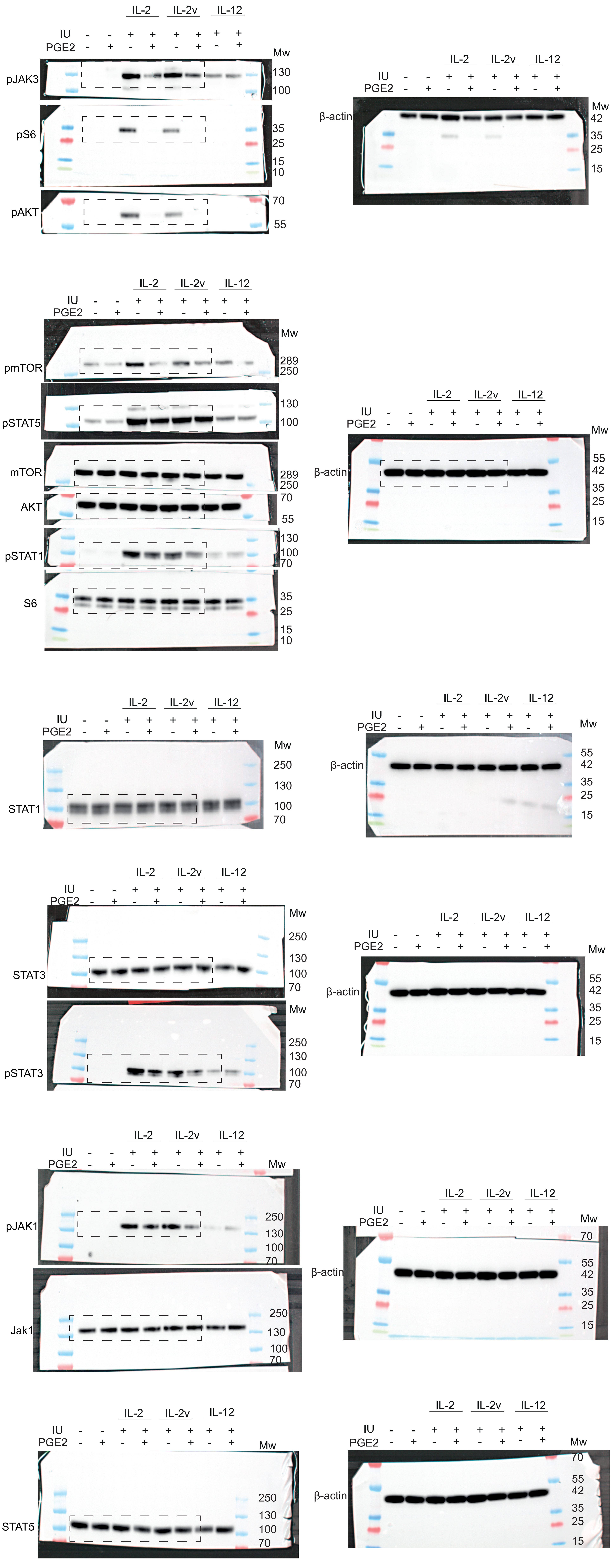

Supplement: Supplementary file 3 — Western blots for IL-2 signalling. Uncut western Blots from IL-2 signalling in RA T cells treated with PGE2 for 48h and subsequently stimulated with IL-2 or an IL-2Rβγc (IL-2v) mutein for 15 min. β-actin controls are depicted on the right. [file 41586_2024_7352_MOESM3_ESM.pdf]
