# Supplementary material for: PGE2 inhibits TIL expansion by disrupting IL-2 signalling and mitochondrial function
Source: Nature. 2024 Apr 24;629(8011):426–34. doi: 10.1038/s41586-024-07352-w (PMC11078736; doi:10.1038/s41586-024-07352-w)

Boxes indicate regions shown in the figure.
Asterisks indicate unspecific bands

In Extended data 5v

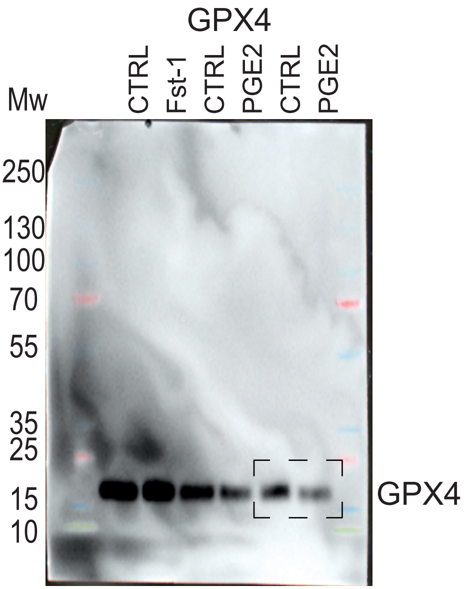

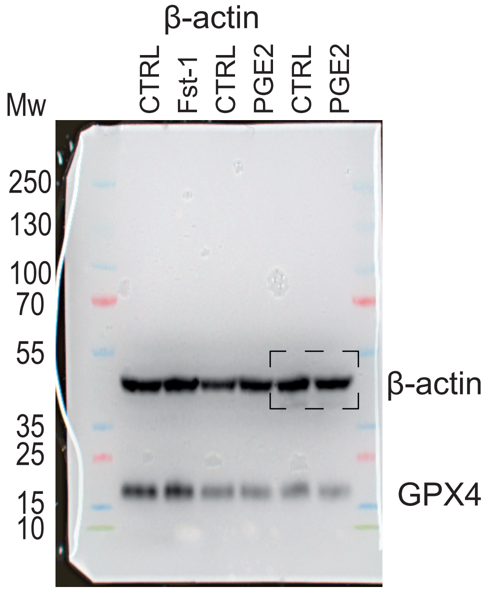

In Extended Data 5i

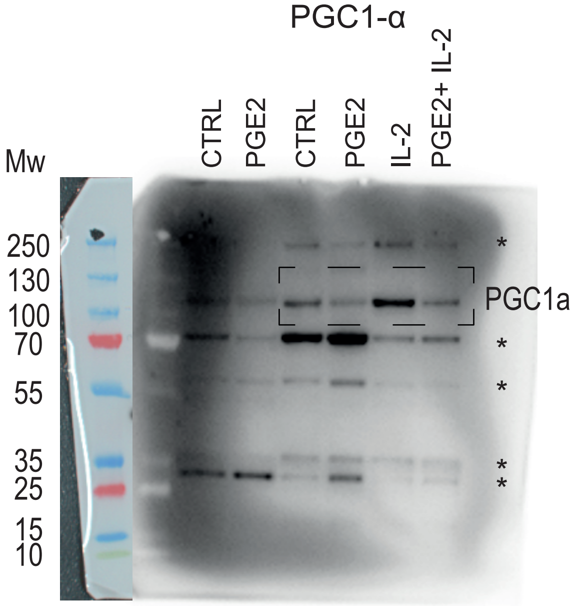

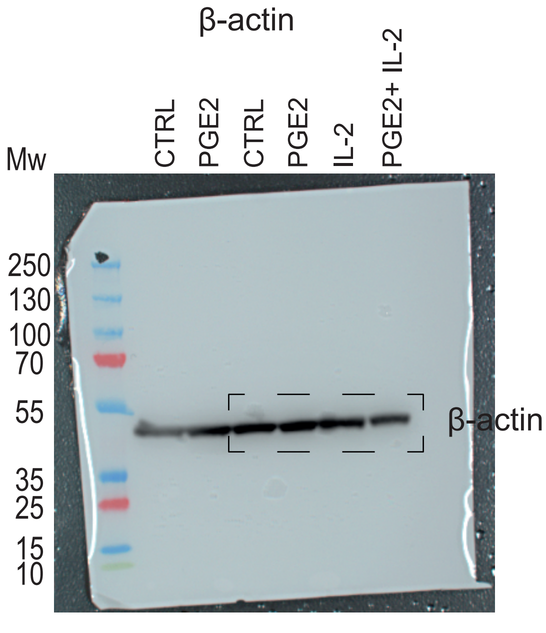

Supplement: Supplementary file 4 — Western blots for PGC1a and GPX4 expression. Uncut western blots from GPX4 protein expression in TILs treated or not with PGE2 or Fst1 (top) and PGC1-α protein expression upon IL-2 stimulation in presence/absence of PGE2 (bottom). β-actin controls are depicted on the right. [file 41586_2024_7352_MOESM4_ESM.pdf]
